# Genome-wide linkage and association study implicates the 10q26 region as a major genetic contributor to primary nonsyndromic vesicoureteric reflux

**DOI:** 10.1038/s41598-017-15062-9

**Published:** 2017-11-03

**Authors:** John M. Darlow, Rebecca Darlay, Mark G. Dobson, Aisling Stewart, Pimphen Charoen, Jennifer Southgate, Simon C. Baker, Yaobo Xu, Manuela Hunziker, Heather J. Lambert, Andrew J. Green, Mauro Santibanez-Koref, John A. Sayer, Timothy H. J. Goodship, Prem Puri, Adrian S. Woolf, Rajko B. Kenda, David E. Barton, Heather J. Cordell

**Affiliations:** 1 Department of Clinical Genetics, Our Lady’s Children’s Hospital, Crumlin, Dublin 12 Ireland; 2 National Children’s Research Centre, Our Lady’s Children’s Hospital, Crumlin, Dublin 12 Ireland; 3 Institute of Genetic Medicine, Newcastle University, Central Parkway, Newcastle upon Tyne, NE1 3BZ UK; 4 UCL Institute of Health Informatics, University College, London, NW1 2DA UK; 5 Department of Tropical Hygiene, Faculty of Tropical Medicine, Mahidol University, Bangkok, 10400 Thailand; 6 Jack Birch Unit of Molecular Carcinogenesis, Department of Biology, University of York, York, YO10 5DD UK; 7 University Children’s Hospital Zurich, Steinwiesstrasse 75, 8032 Zurich, Switzerland; 8 Royal Victoria Infirmary, Newcastle upon Tyne, NE1 4LP UK; 9 University College Dublin School of Medicine, Our Lady’s Children’s Hospital, Crumlin, Dublin 12 Ireland; 10 University College Dublin, Stillorgan Rd, Belfield, Dublin 4 Ireland; 11 Division of Cell Matrix Biology and Regenerative Medicine, Faculty of Biology, Medicine and Health, University of Manchester, Manchester, UK; 12 Royal Manchester Children’s Hospital and Manchester Academic Health Sciences Centre, Manchester, UK; 13 Department of Pediatric Nephrology, University Medical Centre Ljubljana, Ljubljana, Slovenia

## Abstract

Vesicoureteric reflux (VUR) is the commonest urological anomaly in children. Despite treatment improvements, associated renal lesions – congenital dysplasia, acquired scarring or both – are a common cause of childhood hypertension and renal failure. Primary VUR is familial, with transmission rate and sibling risk both approaching 50%, and appears highly genetically heterogeneous. It is often associated with other developmental anomalies of the urinary tract, emphasising its etiology as a disorder of urogenital tract development. We conducted a genome-wide linkage and association study in three European populations to search for loci predisposing to VUR. Family-based association analysis of 1098 parent-affected-child trios and case/control association analysis of 1147 cases and 3789 controls did not reveal any compelling associations, but parametric linkage analysis of 460 families (1062 affected individuals) under a dominant model identified a single region, on 10q26, that showed strong linkage (HLOD = 4.90; ZLRLOD = 4.39) to VUR. The ~9Mb region contains 69 genes, including some good biological candidates. Resequencing this region in selected individuals did not clearly implicate any gene but *FOXI2*, *FANK1* and *GLRX3* remain candidates for further investigation. This, the largest genetic study of VUR to date, highlights the 10q26 region as a major genetic contributor to VUR in European populations.

## Introduction

Primary vesicoureteric reflux (VUR), the retrograde flow of urine from the bladder through the vesicoureteric junction into the upper urinary tract, is the most common renal tract malformation. VUR is usually a benign condition but chronic kidney damage triggered by ascending pyelonephritis and also congenital kidney hypo/dysplasia (collectively known as reflux nephropathy, RN) can occur and lead to end stage renal disease^1,2^. Other congenital anomalies of the kidney and urinary tract (CAKUT) commonly occur along with VUR. The oft-quoted prevalence of VUR is 1–2% but the true prevalence may well be higher^3,4^. The disease has been suggested to be twice as common in females as males^5^ but this most likely reflects an ascertainment bias, and other studies have detected only a slight excess of incidence in females compared to males^6,7^. The prevalence of VUR tends to decrease with age^5^, and serial studies of individual patients show VUR can spontaneously regress during childhood in a subset of initially affected individuals^1,8^. Screening studies of first-degree relatives of individuals with VUR identifies VUR in one third to one half of siblings^9,10^ and 65% of offspring^11^. This observation, coupled with the high concordance of primary VUR in identical twins^12^ and the identification of families with multiple generations affected by primary VUR and RN^13,14^, suggests that there may be a substantial genetic component to VUR. However, large-scale genetic studies of VUR carried out to date have been somewhat disappointing and generally rather inconclusive. Although there have been some compelling findings in individual large families, overall, little concordance is seen between the results from different studies^13–23^, supporting the notion that the condition is genetically heterogeneous. Here we combined data from the two largest genetic studies of VUR conducted to date^19,22^, comprising three separate cohorts (from Ireland, the UK and Slovenia), to investigate whether the increased power obtained from use of a larger sample size could help identify genetic contributors operating across multiple affected families/individuals from these three European populations.

## Results

### Genome-wide Association Analyses

Family-based association analysis carried out using the transmission/disequilibrium test (TDT)^24^ produced no compelling association signals (Supplementary Figure S1), similar to what had been seen previously^19,22^ in individual analysis of the separate cohorts. Case/control analysis of our VUR cases together with population-based controls from Ireland (851 Trinity College Dublin/Irish Blood Transfusion Service BioBank controls)^22^ and the UK (2938 Wellcome Trust Case Control Consortium controls)^19,25^ similarly produced no compelling association signals. We note that the relatively sparse SNP set available for association analysis (see Methods) provides incomplete genome coverage with levels that are probably, at best, close to the 31% coverage provided by the Affymetrix 111 K array^26^. Therefore, our results do not preclude the possibility that common variants associated with VUR exist, but we would need to genotype our UK/Slovenian samples (and ideally further additional samples, including Slovenian controls) with a much denser genotyping array in order to answer this question definitively.

In an attempt to improve genome coverage, we carried out genotype imputation using the Michigan Imputation server^27^ with the Haplotype Reference Consortium (HRC) reference panel^28^, treating the Irish and UK/Slovenian cohorts separately (since they had very different numbers of QCed genotyped SNPs available to inform imputation). We then repeated the TDT association analysis at all SNPs passing post-imputation QC (Supplementary Figure S2); this analysis included 3,600,157 SNPs for the UK/Slovenian cohorts, 6,277,126 SNPs for the Irish cohort, and 3,563,212 SNPs for all three cohorts combined. One region on chromosome 20 showed genome-wide levels of significance (p-value <1 × 10^−8^) when analysis was restricted to the UK/Slovenian cohort, however this association was not replicated in the Irish or combined cohorts (Supplementary Figure S2, Supplementary Table S1) and visualisation of the association pattern in the region using LocusZoom^29^ (Supplementary Figure S3) showed a slightly unusual pattern, leading us to suspect that this may be an imputation artefact. Specifically, a number of SNPs that are known to be highly correlated with the lead SNP rs6138998 (chr20:357700) on the basis of European reference data from the 1000 Genomes Project^30^ (as indicated by their color coding in Supplementary Figure S3) show much lower levels of association (smaller −log_10_ (p-values)) than is seen at the lead SNP, suggesting an inconsistency between results that should in reality be highly concordant. A single SNP on chromosome 6 showed genome-wide levels of significance (p-value <1 × 10^−8^) in analysis of the combined cohorts (Supplementary Figure S2, Supplementary Table S1), but again visualisation of the association pattern in the region (Supplementary Figure S4) showed an unusual pattern, with SNPs that are known to be correlated with the lead SNP rs11759064 (chr6:162800665) showing much lower levels of association than is seen at the lead SNP, leading us to again suspect that this may be an imputation artefact. Given the lack of definitive replication of the chromosome 20 signal and the unusual patterns of association/linkage disequilibrium seen at both signals, we chose not to take either of these findings forward for further interrogation at this time.

### Genome-wide Linkage Analyses

**Figure 1:**
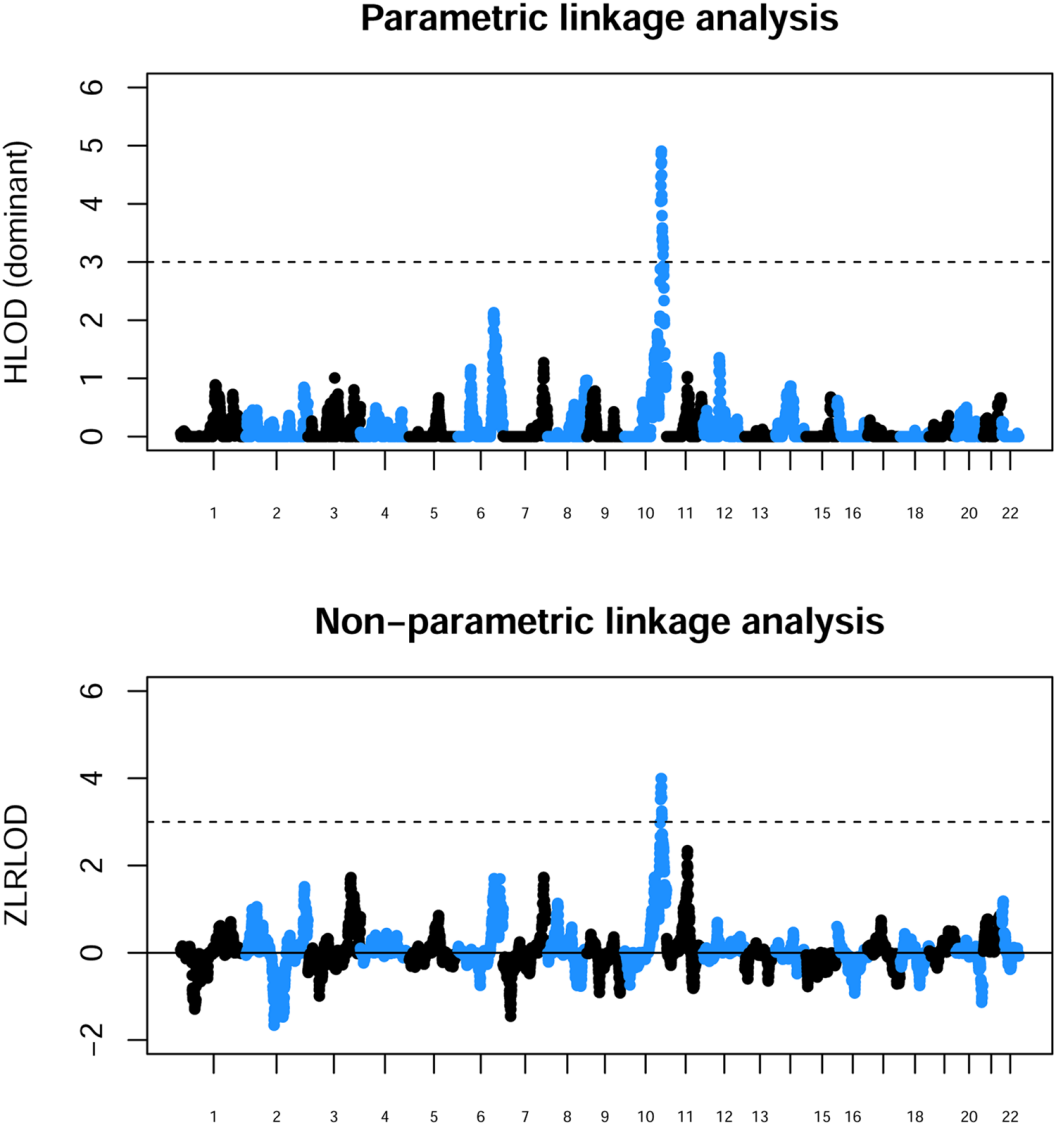
Results from parametric linkage (HLOD) analysis under a dominant model and non-parametric linkage analysis (ZLRLOD) using combined populations. The x axis and alternating colors indicate alternating chromosomes. A dashed line is shown at an HLOD or ZLRLOD threshold of 3.

Parametric linkage analysis under a recessive model gave no strong evidence for linkage at any genomic location (data not shown). However parametric linkage analysis under a dominant model, as well as non-parametric linkage analysis, identified a single genomic location (focused around rs7907300 on chromosome 10q26) showing strong evidence (HLOD = 4.90; ZLRLOD = 4.39) for linkage to an underlying disease locus (Fig. 1), with an estimated proportion (*α*) of linked families of *α* ≈ 30%. This region had shown some evidence of linkage in the previous separate analyses of these cohorts^19,22^ and, indeed, had been highlighted as one of the top findings in the Irish cohort by Darlow *et al*.^22^, although the significance achieved in that previous analysis (HLOD = 2.34, ZLRLOD = 2.06) was not sufficient to definitively conclude the presence of a disease locus in the region. In previous analysis of the UK/Slovenian cohorts^19^ this region had achieved an HLOD of 2.44 and a ZLRLOD of 1.55 in an expanded set of families where affection status was coded as positive for either VUR or RN. Cordell *et al*.^19^ had further noted that the signal of linkage increased to ZLRLOD = 2.32 when analysis was restricted to families where affection status was coded as positive for VUR (regardless of RN status), as in the current analysis.

We investigated the sensitivity of our parametric linkage analysis results to slight changes in the assumed parameters of the underlying dominant model, but found this did not have any substantial effect. In particular, changing the assumed disease allele frequency from 0.001 to 0.01 (as had been used in previous analysis of these data sets)^19,22^, while retaining dominant penetrances (0.01, 0.99 and 0.99), resulted in a slightly increased maximum HLOD of 5.07 at the same genomic location (rs7907300).

**Figure 2:**
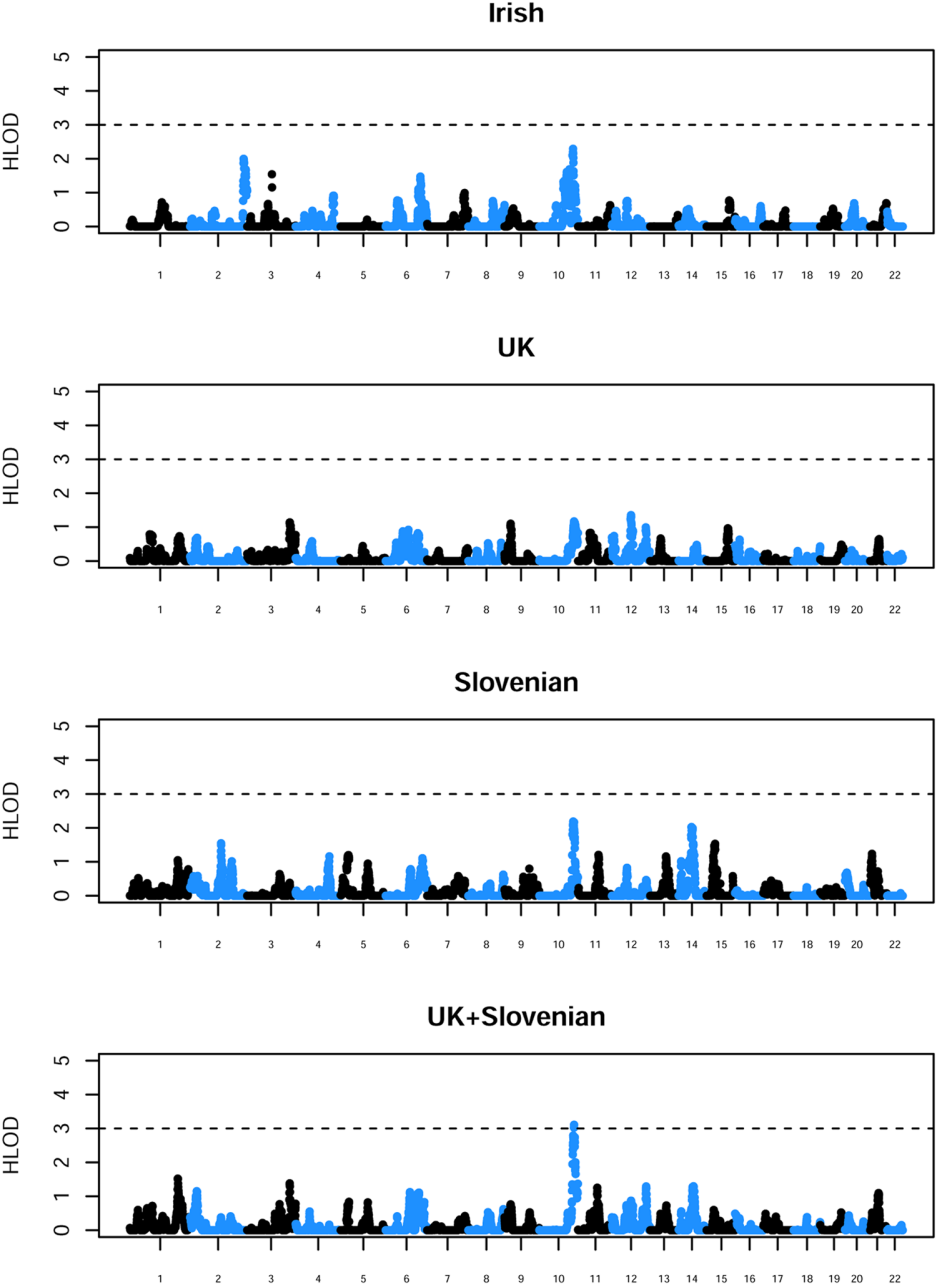
Results from parametric linkage (HLOD) analysis under a dominant model carried out separately within the separate populations. The x axis and alternating colors indicate alternating chromosomes. A dashed line is shown at an HLOD threshold of 3.

To clarify the relative contributions of the individual cohorts to the current results, we repeated the parametric (dominant HLOD) analysis within each cohort separately and within the UK/Slovenian cohorts combined (Fig. 2). The strongest contribution to the 10q26 signal appears to come from the (larger) Irish and Slovenian cohorts, although combining the UK and Slovenian cohorts (as was done previously)^19^ produces a reasonably compelling linkage signal. Neither the individual cohorts nor the UK/Slovenian combined cohort achieve the HLOD of 3.3 that would most probably be required to conclude ‘significant’ linkage^31^ (allowing for the fact that the LOD score has been maximized over two models–recessive and dominant–as well as over a heterogeneity parameter *α*, the proportion of linked families) but the results are certainly consistent with the much stronger (and highly significant) effect seen here when all three cohorts are combined.

**Figure 3:**
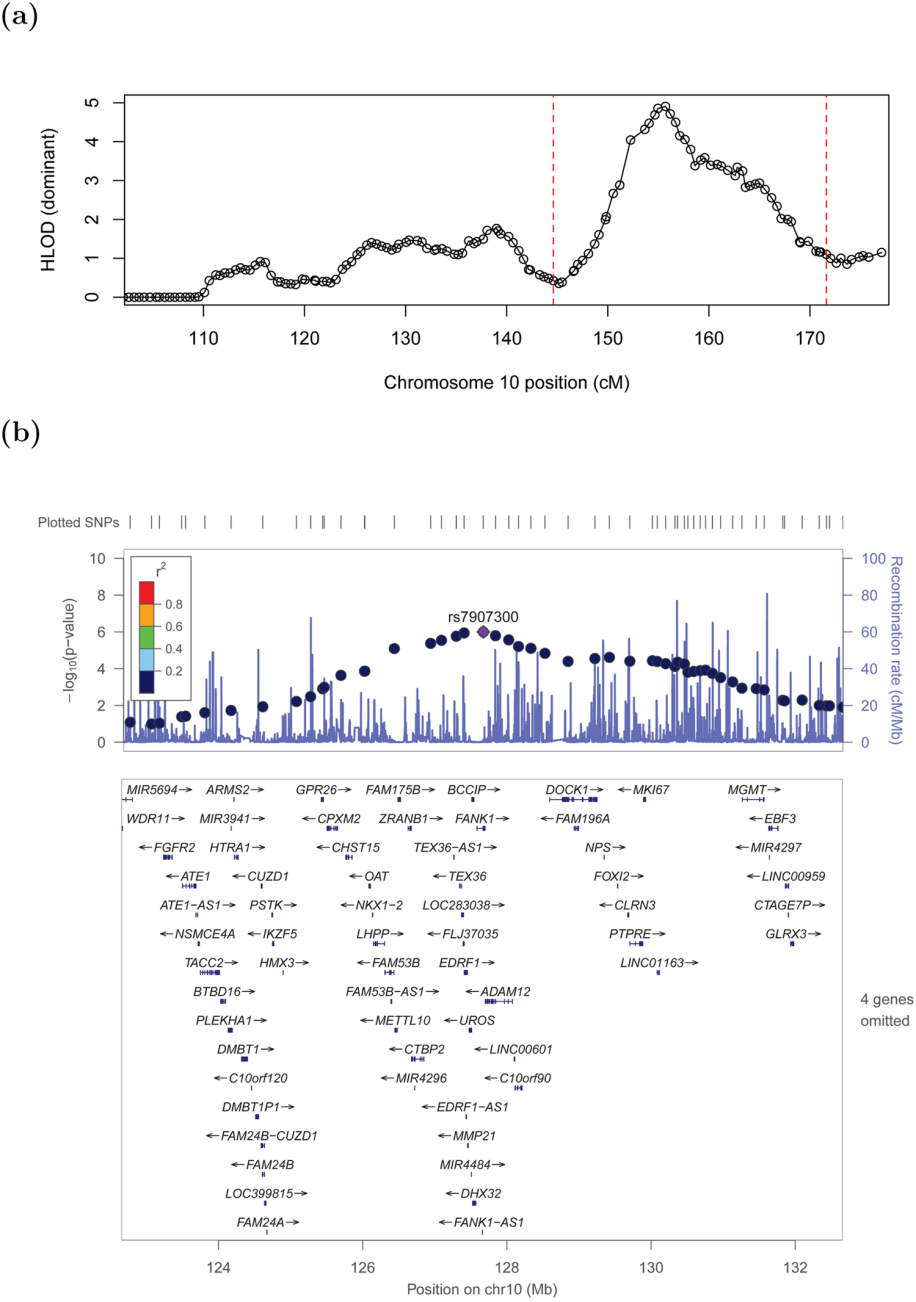
Close-up of the chromosome 10 region displaying the strongest evidence of linkage. (**a**) Results from parametric linkage (HLOD) analysis under a dominant model, (**b**) LocusZoom plot (GRCh37 positions) of the -log base 10 of the HLOD equivalent p-value in the smaller (10 Mb ≈ 27 cM) region (shown within the red dashed lines on panel a) centred around the top SNP, rs7907300. Genes in this region are shown below the plot; the 4 omitted genes all lie to the left of *GPR26* and thus outside the main region of significance.

As is often the case in linkage studies, the plausible region within which the underlying disease lies is very large, containing 44 genes in the central ~7 Mb region or 69 genes in the wider ~9 Mb region (Supplementary Table S2). Figure 3a shows a ‘close-up’ of the signal with circles corresponding to HLOD values at the SNPs used to perform linkage analysis, while Fig. 3b shows a LocusZoom^29^ plot of the equivalent p-values (calculated using the method of Huang and Vieland)^32^ for those SNPs lying within the main peak of significance bounded by red dashed lines in Fig. 3a. (Although these p-values may seem less impressive than those generally seen in LocusZoom plots from genome-wide association studies, we note that the appropriate threshold for declaring genome-wide significance is very different in linkage studies compared to association studies^33^, with genome-wide significance in affected-sib-pair linkage studies corresponding to a p-value of around 2.2 × 10^−5^). The 4 genes omitted from the LocusZoom plot in Fig. 3b (*C10orf88*, *ACADSB*, *HMX2* and *BUB3*) all lie to the left of (proximal to) *GPR26* and thus would probably not be considered good candidates for harboring causal mutations, given the decrease in linkage signal observed within this section of the plot, but any of the genes to the right of *GPR26* might be considered good positional candidates.

### Regional Association Analyses

To investigate evidence for allelic association within the implicated 10q26 linkage region, we examined more closely our results from HRC imputation (followed by TDT association analysis) within the 12 Mb region spanning the linkage signal (Supplementary Figures S5–S7). In the Irish cohort (Supplementary Figure S5), a suggestive association signal (p-value 2.1 × 10^−6^) is seen at around 129.5 Mb (Build 37). This is not replicated in the UK/Slovenian cohorts (Supplementary Figure S6), however further inspection revealed that these SNPs had not passed post-imputation QC in the UK/Slovenian cohorts, on account of imputation Rsq values ≈0.66. When we retain these SNPs for analysis (taking the view that Rsq values of 0.66 represent a reasonable level of imputation quality) they do not, in fact, replicate the Irish findings (Supplementary Table S3): only nominal levels of significance are reached with the transmission patterns (and odds ratios) going in the opposite drection from that seen in the Irish cohort. We also note that this Irish signal lies some distance away from the location of the top linkage signal (at 127.7 Mb) and is thus anyway unlikely to be a strong contender for explaining the linkage signal.

In the UK/Slovenian cohorts (Supplementary Figure S6), association signals are seen at 126.0 Mb (p-value 1.1 × 10^−6^), 127.6 Mb (p-value 8.7 × 10^−6^), 128.4 Mb (p-value 6.1 × 10^−6^) and 131.5 Mb (p-value 2.9 × 10^−7^). However these signals are not replicated in the Irish cohort (Supplementary Figure S5, Supplementary Table S3). Moreover, only one of these signals lies close to the location of the top linkage signal. When the combined cohorts are analysed (Supplementary Figure S7), no SNPs pass the suggestive significance threshold of p-value 1.0 × 10^−5^).

The weak/inconsistent evidence of association seen within the 10q26 linkage region suggests that common alleles are unlikely to be contributing to the linkage signal; indeed this scenario would seem a priori unlikely given the underlying Mendelian dominant model (operating in ≈30% of families) that was used to generate the linkage signal in the first place. A far more likely scenario is that the linkage signal is caused by heterogeneous mutations operating within different families in a dominant or dominant-like fashion. Given that the TDT can be considered as a test of linkage in the presence of association (particularly when applied to non-independent affected sibs, as here) we conclude that the apparent TDT association signals seen most likely reflect linkage between alleles at the relevant SNPs and mutations at the underlying disease locus, coupled with perhaps low levels of allelic association (linkage disequilibrium) between alleles at these loci in the founders.

### Exome sequencing within 10q26 region

To interrogate in more detail the genes in the implicated 10q26 region, we performed whole-exome sequencing in a subset of our families. Next-generation sequencing has been extremely successful for the detection of disease-causing mutations in Mendelian, monogenic disorders^34–40^, especially when applied to multiple affected individuals originating from the same family. A caveat in relation to our own study, however, is that we expect that only around 30% of our families will actually harbor mutations at the disease-causing genetic element in the 10q26 region (and, indeed, the accuracy of this estimate of 30% depends on the parameters of the underlying assumed dominant disease model being approximately correct). Prioritizing affected individuals from families where affected sibs shared alleles IBD, we whole-exome sequenced 29 individuals from the 8 families (two UK, two Slovenian, four Irish) showing the strongest evidence of linkage in the 10q26 region. There were 37,930 non-synonymous variants called across the whole exome in the 29 individuals. Of these, 139 occurred within the 10q26 region of interest and 37 of these were relatively rare (allele frequency <0.05) in the 1000 Genomes and Exome Sequencing Project data sets. Of these relatively rare variants, 23 were found within the same gene in two or more individuals (for 7 different genes) and 6 were predicted to be deleterious by at least one predictor out of SIFT/PolyPhen/MutationTaster^41–43^ (Supplementary Table S4). However, further interrogation of the sequences indicated that only one of these genes, *FANK1*, harbored rare variants that were consistent with the IBD sharing and inheritance patterns seen within multiple families (Supplementary Table S5). Further investigation of *FANK1* using 189 European sequences from the 1000 Genomes Project identified 715 variants, of which 407 were ‘rare’ (alternative allele frequency AAF < 0.05). Only 7 of the 189 1000 Genomes Project sequences had no rare variants at all, suggesting that the detection of rare variants in *FANK1* in our own VUR cases is not, in fact, a rare event, and so may not necessarily be related to their phenotype. Moreover, given a VUR population prevalence of up to 3%, we note that the ‘rare’ *FANK1* variants listed in Supplementary Table S5 are actually much too common to realistically be considered as causes of VUR, without invoking a model involving extreme reduced penetrance.

### Targeted sequencing within 10q26 region

Given that we did not find any fully convincing causal variants for VUR within the coding parts of the 10q26 region (in the 8 families we examined), a plausible explanation is that such variants may be found within non-coding, regulatory regions. To investigate this possibility, we performed targeted genomic resequencing of the implicated 9 Mb region of 10q26 (from 123 Mb–132 Mb) in 32 unrelated affected individuals from those families in our data set that contributed the most to the linkage signal (the 8 families that had been whole-exome sequenced and 24 others). Of the 34,412 single nucleotide variants (SNVs) passing standard quality control steps, we identified 9170 that were either absent or present at less than 1% in the 1000 Genomes European data, 3625 of which were also shared between affected individuals (i.e. were present in at least two of the 32 individuals sequenced). We consider this large number of SNVs identified as showing frequency differences between our samples and the 1000 Genomes European samples as most likely to be artefacts arising from the systemic differences in read depth, alignment and variant-calling pipelines between cases and controls. Concentrating on rare SNVs found within promoters in our 9 Mb target region, we found that 69 of 80 promoter-region SNVs occurred within the promoter of *FANK1*, while 11 occurred within the promoter of genes other than *FANK1* (Supplementary Table S6). Arguably the most interesting of the non- *FANK1* genes is *GLRX3*, which harbors a rare haplotype seen in 8 out of our 32 VUR cases. However, the location of *GLRX3* at the far end of the implicated 9 Mb region, distal to the main peak of linkage (Fig. 3), argues against it being a strong positional candidate for explaining the linkage signal. In addition, inspection of the sequencing reads in the relevant 8 VUR cases indicates that the alternative alleles are only seen in the first or last 25 bp of any given read, and only in ~15% of high-quality reads, which may be cause for caution. However, it would certainly be interesting to attempt to validate these observations in these 8 VUR cases using alternative sequencing methods, and, assuming that they validate, to further investigate whether rare variants in *GLRX3* occur in a significant proportion of our VUR cases.

With respect to *FANK1*, further interrogation of the .bam files showed that the *FANK1* region appears to be hypervariable and many of the called SNVs were clearly artefacts (Supplementary Figure S8). After extensive investigation, only 11 of the 69 *FANK1* SNVs appeared potentially genuine (Supplementary Table S7). These included a G/C SNV at 127,584,687 bp and an immediately adjacent C/A SNV at 127,584,688 bp which appeared as heterozygous in all 30 (out of the 32) individuals in which it was called. The variant calls appeared genuine, however we interpret these results with caution since, from our linkage analysis, we are only expecting putative causal variants to be shared in around 10 to 15 of our samples; the sharing of the same causal variant by 30 affected individuals from 30 different families seems somewhat suspicious.

Our suspicions were confirmed when we performed a BLAST search on the 500 bp sequence around these SNVs, and found that the sequence containing the alternative C and A alleles at 127,584,687 and 127,584,688 bp respectively showed high levels of sequence similarity to the short arm of chromosome 22 on the GRCh38 build of the genome (which is unmapped in GRCh37), as well as to the desired 10q26 region. This observation led us to re-align our targeted sequences to the GRCh38 assembly. 9 of the 11 previously identified SNVs in the *FANK1* promoter region were found to align fully to chromosome 22, with only the two at 127,584,687–127,584,688 bp (125,896,118–125,896,119 bp on GRCh38) mapping to chromosome 10. This suggests that a number of the reads identified by our pipeline as mapping to 10q26 may in fact really come from chromosome 22, offering a possible explanation for the unusually high level of variability observed (Supplementary Figure S8).

Further investigation revealed that the 42.4 kb region of chromosome 10 from 125,885,884 to 125,928,375 contains segmental duplications from at least 4 other genomic locations (Supplementary Figure S9). We consider that these segmental duplications are most likely to have generated the high proportion of rare variants recorded for *FANK1* and its promoter, which therefore represent artefactual findings unrelated to the VUR phenotype. More sophisticated experimental methods (such as amplification via long-range PCR prior to sequencing, or the use of longer-read sequencing technologies) will therefore be required in order to robustly interrogate this 42.4 kb region, without danger of contamination from other genomic regions.

### TASER analysis

The analyses described above compared observed allele frequencies in our VUR cases to those in publicly available summary data provided by the 1000 Genomes Project (and similar resources), in order to identify putative causal variants. To achieve a better comparison between our sequenced VUR cases and phenotypically normal controls, we obtained the .bam files from 1106 ALSPAC controls^44^ sequenced as part of the UK10K study^45^. Data sets such as UK10K are usually sequenced at low coverage (typically 2–20 fold) whereas our own sequences have up to 1000 fold coverage in the target region. Analysis of sequence data where there are systemic differences in coverage between cases and controls typically leads to inflated type I errors^46^, but discarding those samples with insufficient read depth can result in a loss of power (or, in this case, the loss of all controls). We therefore used the software package TASER^47^, which effectively re-calls variants in both cases and controls, allowing for systematic differences in read depth (and other factors), at the same time as constructing a test statistic.

In an adaptation of the original implementation of TASER, which splits the entire genome into small windows of interest (corresponding, for example, to genes or exons of genes), we considered our entire target region to be ‘of interest’. For each of the 1106 ALSPAC control sequences and 32 VUR case sequences, we split the target DNA sequence into consecutive 120 bp windows with 30 windows included within a processing ‘block’. This resulted in 2490 blocks covering the entire sequence from 121,240,479 bp to 130,201,779 bp (GRCh38) on chromosome 10. Only bases called with a quality score >30 were added to the read count at each position. We found that only counting alleles that are seen at least twice in the low depth control sequences and at least 5 times in the high depth VUR sequences (in order to reduce the expected number of sequencing artefacts) improved the overall distribution of test statistics (Supplementary Figure S10), which, nevertheless, were slightly lower than expected under the null hypothesis (genomic control factor^48^ *λ* = 0.76). With the MAF filtering threshold set to 0.05 in the base population, we obtained a few signals that were mildly suggestive of association but nothing that reached experiment-wide significance. The output of the current implementation of TASER does not indicate the position of the variants contributing to the test statistic within each window, but by inspecting the input and .bam files we were able to identify the probable variant driving the signal and discount any signals caused by sequencing error (3 out of the top 10 signals). The top findings (Supplementary Table S8) included two rare SNPs (rs183471950 and rs541040960) in two different introns of *TEX36*, a single T insertion in an intron of *DOCK1* (rs551248465), a SNP approximately 3 kb upstream of *FAM175B* (rs533072490) and a further three SNPs in the intergenic region between *LINC01163* and *MGMT*: rs146303503, rs182100352 and an unknown G/A substitution at position 128770839. Given the relatively low frequencies of all these variants seen within the VUR cases, and the relative lack of statistical significance obtained (given the overall number of windows tested), we do not consider any of these results particularly compelling.

In addition to the findings listed in Supplementary Table S8, we found that the windows spanning the 42 Kb segmental duplication region around *FANK1* showed high levels of variation within both cases and controls, offering yet more evidence that the putative causal variants identified by exome and targeted sequencing are almost certainly artefacts derived from amplification of highly similar sequence from multiple chromosomes. Further investigation of this region in selected samples via long-range PCR has identified several more currently undescribed segmental duplications, in addition to the four already discussed (data not shown). Thus this region, containing the key candidate gene *FANK1*, remains effectively untargeted by our current sequencing endeavours, due to our inability to reliably map the sequence reads in this region.

### Gene expression in Normal Human Urothelial (NHU) cells

**Figure 4:**
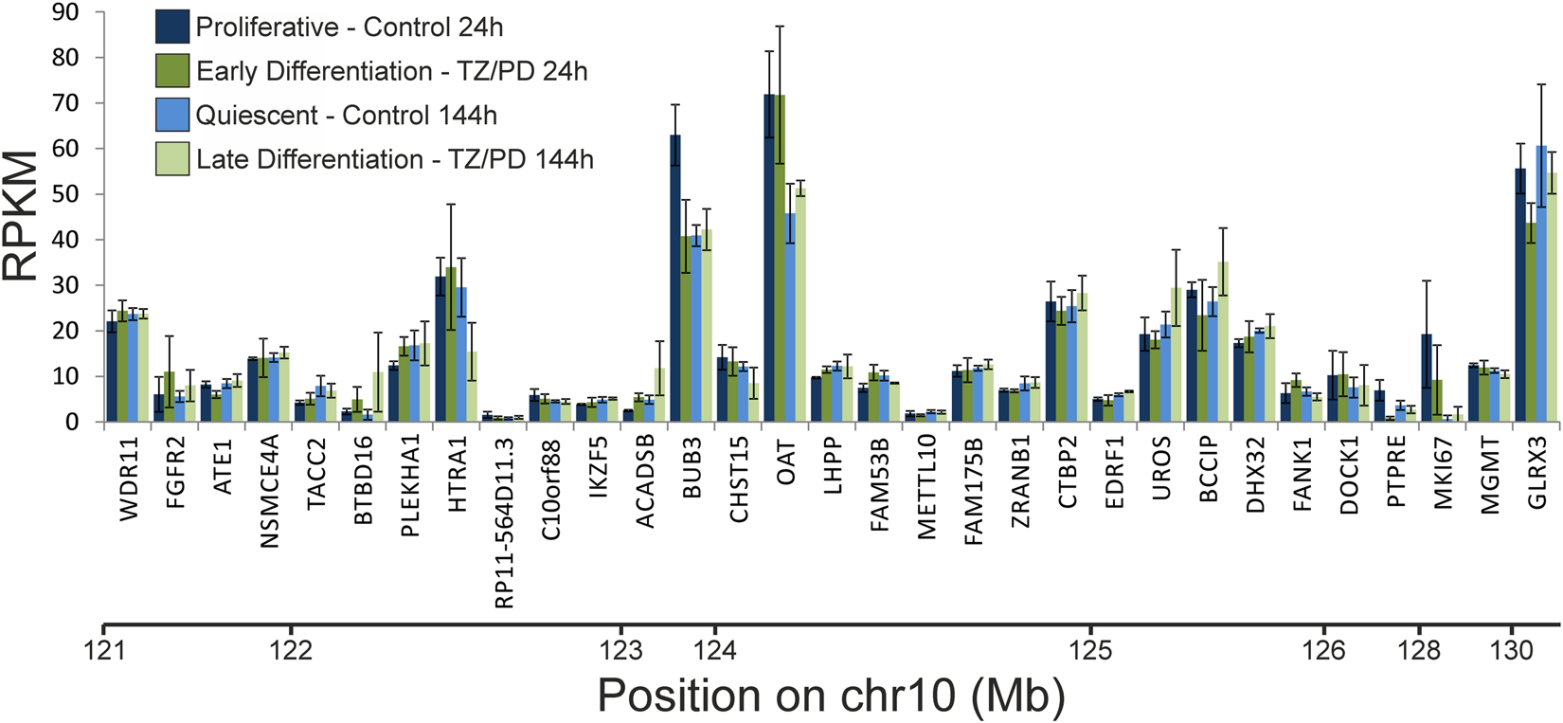
Transcript expression by *in vitro*-propagated cultures of Normal Human Urothelial (NHU) cells mapped to position along chromosome 10q26 (GRCh38 assembly); non-expressed (RPKM ≤ 1 in Control 24 h) genes not shown. Data from RNA-seq expressed as Reads Per Kilobase of transcript per Million mapped reads (RPKM), with each bar representing the mean (±sd) from three independent donor cell lines, as detailed in Fishwick *et al*.^49^.

To help further prioritize genes in the implicated chromosome 10q26 region, data on gene expression by *in vitro*-propagated cultures of Normal Human Urothelial (NHU) cells in different differentiation states were obtained from Fishwick *et al*.^49^. Results (Fig. 4) indicated relatively high levels of gene expression of several genes of interest including *BUB3*, *OAT* and *GLRX3*–although, as mentioned previously, the locations of *BUB3* and *GLRX3* (outside the main peak of linkage) argue against these being strong positional candidates for explaining the linkage signal. *OAT*, the only one of the three highest expressed genes in normal urothelium to be moderately near to the centre of the linkage region, encodes the mitochondrial enzyme ornithine aminotransferase, so is an unlikely candidate on grounds of function.

## Discussion

Here we have identified, through genome-wide parametric and non-parametric linkage analysis, a region on 10q26 that shows strong evidence of harboring genetic variants contributing to primary nonsyndromic VUR. As is often the case in linkage studies, the plausible region in which the underlying disease locus must lie is very large, and, despite our attempts via association analysis and targeted sequencing (in a subset of samples), we have, as yet, been unable to find variants within the region that plausibly account for the linkage signal observed, although the promotor of *GLRX3* remains a key region for further investigation. Moreover, given our discovery that the 42.4 kb region of chromosome 10 from 125,885,884 to 125,928,375 contains segmental duplications from other genomic locations, this region (which contains the key candidate gene *FANK1*) remains effectively untargeted by our current sequencing endeavours. More sophisticated methods (such as amplification via long-range PCR prior to sequencing, or the use of longer-read sequencing technologies) will be required in order to robustly interrogate this region, without danger of contamination with sequence from other genomic regions.

There are five genes on chromosome 10 that are already known to be involved in urinary tract development^22^, four of which lie close to the 10q26 region implicated here, in addition to another, as yet unidentified, gene near the 10q telomere^50^. However, these previously-implicated genes lie mostly outside our main peak of linkage. According to the gene expression atlas GUDMAP (GenitoUrinary Development Molecular Anatomy Project)^51,52^, mouse homologues of most of the human genes in the critical Chr10 locus are normally expressed in at least one site in the developing murine kidney and urinary tract. It is, however, of particular note that: *Cpxm2* is expressed in the mature ureter and bladder; *Fam53* is expressed in the developing and mature ureter; *Dhx32* is expressed in the developing and adult ureter and bladder; *Adam12* is expressed in the developing ureter and bladder; and *Ptpre* is expressed in the developing and adult ureter. Expression during *in vitro* differentiation of normal human urothelial cells might be considered a more relevant factor than mouse gene expression data obtained from GUDMAP, and indeed there are proven human VUR genes that encode tenascin XB and uroplakins which are expressed in the urothelium^23,53,54^. Cross-referencing the genes in our implicated 10q26 region with expression data from normal human urothelial cells^49^ highlighted several genes of interest including *BUB3*, *OAT* and *GLRX3*. Of these, only *OAT* would seem a strong positional candidate, but is a highly unlikely candidate on grounds of function. Our other key candidate, *FANK1*, was also expressed, albeit at a low level. However, this analysis of human urothelial cells comes with the caveat that epithelium is just one type of tissue found in the urinary tract, and we do not currently have similar data for other components such as nerve and muscle/mesenchyme.

Arguably the best candidate gene in the region is *FGFR2*, which has been implicated in induction abnormalities of the ureteric bud and development of VUR in mice^55–58^. However, the localisation of *FGFR2* outside our main peak of linkage argues against it being a strong candidate for harboring the mutations that contribute to the linkage signal detected in our families. It is worth mentioning that *FGFR2* is involved not only in the embryonic development of the metanephric urinary tract but also in that of the skeleton and skin, and mutations in the gene in humans can cause Crouzon syndrome, Pfeiffer syndrome, Apert syndrome, Jackson-Weiss syndrome, Beare-Stevenson cutis gyrata syndrome, or Saethre-Chotzen syndrome. Demonstration of VUR in mice^58^ was achieved by conditional knock-out of *Fgfr2* very specifically in the peri-Wolffian duct stromal cells only, and therefore any mutation causing non-syndromic VUR or other CAKUT in humans that affects *FGFR2* is likely to be in a non-coding regulatory element that affects expression of the gene in the developing urinary tract but has no effect on its expression in other tissues. Since most, if not all, genes already known to be involved in urinary tract development are also known to be involved in the development of other structures, including the eye, the ear, the branchial clefts (and structures that develop in them, such as the thyroid gland) the skeleton and the gut, it is quite possible that most mutations that cause VUR will be regulatory mutations and in non-coding DNA, whatever gene they affect.

A stronger positional candidate, given the linkage evidence, is *FOXI2*. GUDMAP shows that *Foxi2* is very highly expressed in the mouse metanephric mesenchyme at the appropriate time to stimulate the growth of the ureteric bud. However, our investigation of expression in human urothelial cells failed to detect *FOXI2* expression. In 2009 the Irish group used Sanger sequencing to explore this gene, including all the untranslated sequence, 1 kb of promoter sequence upstream, and several highly conserved non-coding regions in the gene desert at distances up to 1 Mb away, in 232 VUR index cases. Eleven of the variants found in the conserved intergenic elements had ‘Restricted Substitution’ (GERP) scores >4, of which four have still not appeared in dbSNP, and, of these, one is immediately adjacent to a predicted POU3F1 binding-site and another is within a predicted PRRX2 binding-site. *Pou3f1* and *Prrx2* are both expressed in mouse metanephric mesenchyme, the latter very highly. However, functional experiments would need to be carried out to determine whether any of the variants actually affect expression and, if so, which gene is affected. *FOXI2* itself was sequenced at that time in case there might be a mutation in the gene which would help to confirm its candidacy. Only two novel variants with GERP scores >2 were found in the gene, neither of which were likely pathogenic, and there was not an opportunity to pursue investigation of the intergenic variants at that time. Sequencing right across the whole linkage region with a larger number of patients (and controls) should hopefully enable identification of the correct regulatory element(s), but it is still very likely that functional testing will be required to determine which gene is affected.

In conclusion, this, the largest genetic study of VUR to date, provides strong evidence for the 10q26 region as the major genetic contributor to primary nonsyndromic vesicoureteric reflux in European populations. Definitive identification of the causal variants contributing to this observation will be required in order to identify the mechanism involved. Removal of families with pathogenic mutations in this region (once identified) from the data set should make it easier to identify, by reanalysis, the next most important of the many loci expected to be responsible for VUR in the 70% or more of families that are not linked to 10q.

## Methods

### Samples and Genotyping

We combined data from the two largest genetic studies of VUR conducted to date^19,22^. Full details of sample collection, phenotype definition and genotyping can be found in the primary publications from these studies^19,22^. In brief, one study^22^ consisted of 235 Caucasian families (500 affected individuals) collected from two hospitals in Dublin, Ireland. Families with two or more affected members with primary VUR of any grade were collected; the majority of the families were nuclear (affected-sib-pair) families containing two siblings with VUR, with genotype data available for siblings and both parents. Six family-members (three parents and three siblings) of VUR patients who had CAKUT (without detected VUR) were also counted as affected. The other study^19^ included two separate cohorts, one from the UK and one from Slovenia. The UK cohort comprised 165 Caucasian families (303 affected offspring) collected from multiple centres across the UK; similar to the Irish cohort the majority of the families were nuclear (affected-sib-pair) families containing two siblings with VUR and with genotype data available for siblings and both parents. The Slovenian cohort comprised 148 Caucasian families (353 affected individuals, 313 affected offspring) collected from the University Medical Centre, Ljubljana; again the majority of the families were nuclear (affected-sib-pair) families containing two siblings with VUR, with genotype data available for siblings and both parents.

The classical grading system for VUR^59^ involves classifying the disease into (increasingly severe) grades from I-V according to the site and severity of reflux based on the degree of filling and dilation of the ureter and upper urinary tract, as assessed by cystography. In the Irish cohort, VUR was diagnosed via micturating cystourethrograms allowing VUR grade to be determined in 96% of patients. In the UK/Slovenian cohorts, however, we elected at the start not to include this concept in the analyses. This was because a. the classic grading system was not in fact always used in the micturating cystogram reports and b. the same grading system is not generally used for radioisotope cystograms, a techique increasingly used to diagnose VUR. Thus, for the analyses described here, we considered VUR to be radiologically proven, but did not consider its grade.

Because of sample drop-out, affection status and family structure, not all samples/families constributed to all analyses. We estimated that 199 Irish families (467 affected individuals), 121 UK families (258 affected individuals) and 140 Slovenian families (337 affected individuals) contributed to the linkage analyses presented here. Slightly larger numbers of families/individuals contributed to association analysis (family-based and case/control), as association analysis could potentially make use of family structures that were uninformative for linkage (such as families where only one affected individual remained, following strict quality control (QC) procedures). The female:male gender ratio for patients used in the genetic analyses was 1.49 in the Irish cohort, 1.29 in the UK cohort (slightly higher than the 1.21 ratio seen in the full UK cohort)^60^ and 2.04 in the Slovanian cohort. We chose not to reduce our sample size by stratifying the analysis by gender, which for genetic studies would in any case only be likely to have any substantial effect with regards to variants on the X chromosome. We also chose not to formally account for age of diagnosis in the analysis, taking the view that for genetic studies the most relevant measure is simply the fact that an individual is affected, and whether this can be related to their genotype or shared genotype with other affected relatives. However, given that the prevalence of VUR decreases with age (on account of spontaneous resolution), we counted siblings of affected children who were not investigated until later ages as unknown for VUR affection status, if VUR was not found.

The UK/Slovenian study, intitially conceived as a linkage study, made use of a relatively sparse genotyping array, the Affymetrix NspI array, containing 262,264 genome-wide SNPs. Following ‘medium-level’ QC checks^19^, 134,350 SNPs in the UK and Slovenian cohorts remained available for analysis. The Irish cohort was genotyped on the on the Affymetrix Genome-Wide Human SNP Array 6.0, containing 834,482 genome-wide SNPs. Following QC checks^22^, a final set of 643,691 SNPs in the Irish cohort remained available for analysis. The density of the final set of SNPs available is more than sufficient for genomewide linkage analysis (for which only 2–3 SNPs of high minor allele frequency per cM are required) in all three cohorts; however, for genomewide association analysis, the UK and Slovenian cohorts are most likely underpowered with respect to providing full genome coverage, as illustrated by the fact that in imputation analysis (see below) only 3,600,157 SNPs were successfully imputed for the UK/Slovenian cohorts, in comparison to 6,277,126 SNPs for the Irish cohort.

### Genome-wide Association Analyses

Family-based association analysis using the transmission/disequilibrium test (TDT)^24^, implemented in the program PLINK^61^ and applied to all affected sibs (assumed to be independent, conditional on parental genotype), was carried out at the subset of 119,548 genotyped autosomal and X-chromosomal SNPs passing quality control checks in all three cohorts. (TDT analysis within the separate cohorts and in the combined UK/Slovenian cohort had been carried out previously^19,22^, without detecting any compelling (e.g. genome-wide significant) associations or any significant asociations that replicated across different cohorts).

We also performed case/control analysis of our VUR cases together with population-based controls from Ireland (851 Trinity College Dublin/Irish Blood Transfusion Service BioBank controls)^22^ and the UK (2938 Wellcome Trust Case Control Consortium controls)^19,25^ at 108,134 autosomal SNPs passing QC in all case and control cohorts. This analysis was carried out using a linear mixed modelling approach implemented in the software FaST-LMM^62^ in order to adjust for both relatedness between cases and population differences between cases and controls^63–66^.

### Genome-wide Linkage Analyses

We carried out multipoint parametric and non-parametric linkage analysis on the combined Irish/UK/Slovenian cohorts using the software packages MERLIN and MINX^67^. To construct a suitable SNP set for multipoint linkage analysis, we first pruned our data set to include only those SNPs with minor allele frequencies greater than 0.3 and showing low levels of inter-marker linkage disequilibrium (using the PLINK command “–indep 50 5”), and then thinned the data to use only the two SNPs with the highest heterozygosity in each 1 cM window, using the program MapThin (http://www.staff.ncl.ac.uk/richard.howey/mapthin/). Information content plots from MERLIN and MINX indicated that the resulting SNP set (containing 6585 autosomal and 337 X-chromosomal SNPs) achieved high levels of information content (≈95%) across the entire genome (data not shown).

Parametric linkage analysis allowing for heterogeneity (an “HLOD” analysis) was carried out assuming a disease allele frequency of 0.001 and either a recessive (penetrances 0.01, 0.01 and 0.99) or dominant (penetrances 0.01, 0.99 and 0.99) model. (Results were found to be not too sensitive to small perturbations of these assumed disease allele frequencies and penetrances). We chose to assume a slightly rarer disease allele frequency (0.001) than the 0.01 that had been used in previous (separate) analyses of these data sets, in order to better match our expectation that the disease is highly genetically heterogeneous and so the population frequency of any particular causal genetic variant is likely to be small. Non-parametric linkage analysis was carried out using the equivalent LOD score to the Kong and Cox exponential model likelihood-ratio allele-sharing test^68^, which we denote here as “ZLRLOD”.

### Imputation Analysis

To improve genome coverage, allowing interrogation of a denser set of SNPs, we carried out SNP imputation followed by TDT association, treating the Irish and UK/Slovenian cohorts separately (since they had very different numbers of QCed genotyped SNPs available to inform imputation). We used the Michigan imputation server^27^ with the Haplotype Reference Consortium (HRC) reference panel^28^ and pre-phasing performed via Eagle2^69^. ‘Best-guess’ imputed genotypes were used, subject to the posterior probability of the genotype call exceeding 0.9. To ensure that only high-quality imputed SNPs were included in the final set, imputed SNPs were filtered to include only those with minor allele frequency >0.02 and imputation Rsq >0.8.

### Exome sequencing within 10q26 region

We whole-exome sequenced 29 individuals from the 8 families (two UK, two Slovenian, four Irish) showing the strongest evidence of linkage in the 10q26 region. Sequencing was carried out by AROS using Illumina’s Nextera Rapid Capture Exome Kit followed by 100 bp paired end sequencing (6 samples per flow cell lane) on the Illumina HiSeq 2000/2500 platform. Sequences were aligned to the Human GRCh37 assembly using BWA^70^ and variant calling was performed with the GATK HaplotypeCaller algorithm following best practice recommendations^71–73^. Sequencing reads were examined via visualisation of the .bam files in IGV^74^.

### Targeted sequencing within 10q26 region

We performed targeted genomic resequencing of the implicated 9 Mb region of 10q26 (from 123 Mb-132 Mb) in 32 unrelated affected individuals from the families in our data set that contributed most to the linkage signal. Sequencing was carried out by Oxford Gene Technology (OGT) (whose next generation sequencing services have now been transferred to Source BioScience) using SureSelectXT Custom 9 Mb design and capture followed by 100 bp paired end sequencing (11 samples per flow cell lane) on the Illumina HiSeq 2000 platform. Sequences were again aligned to GRCh37 with BWA, variants were called with GATK HaplotypeCaller following best practice recommendations, and reads were visualised via IGV^74^.

### TASER analysis

We analysed the targeted resequencing data from the 32 unrelated affected individuals together with whole-genome sequence data from 1106 ALSPAC controls^44^ sequenced as part of the UK10K study^45^. We used the software package TASER^47^, which effectively re-calls variants in both cases and controls, allowing for systematic differences in read depth (and other factors), at the same time as constructing a test statistic. TASER uses the total number of reads mapped to a variant, and the number carrying the minor allele, to calculate a score statistic at each position in a gene (or window) of interest, thus providing an assessment of the effect of each individual variant on the disease phenotype. A burden statistic is then calculated for each window as the sum of the score statistics for each of the variants within that window, allowing identification of windows that have a higher or lower accumulation of rare variants in the cases than might be expected, compared to controls. This procedure therefore effectively looks for windows containing clusters of rare variants seen in different affected individuals (and not seen - or at least not at comparable frequencies - in controls).

### Gene expression in Normal Human Urothelial (NHU) cells

Data on gene expression by *in vitro*-propagated cultures of Normal Human Urothelial (NHU) cells were obtained from Fishwick *et al*.^49^ for the genes in the implicated chromosome 10q26 region (Supplementary Table 1). Transcript expression was measured via RNA-seq. NHU cells were maintained as finite, serially-passaged cell lines in keratinocyte serum-free medium containing bovine pituitary extract and epidermal growth factor and supplemented with 30 ng/ml cholera toxin. Results were obtained for non-differentiated (vehicle control 0.1% DMSO) cultures in proliferating (24 h) and quiescent, contact-inhibited (144 h) states and at parallel time-points (24 h and 144 h) following induction of differentiation. Differentiation was induced using 1 *μ*M troglitazone (TZ) as PPAR *γ* ligand with concurrent 1 *μ*M PD153035 to block epidermal growth factor receptor activation. Change in expression of MK167 was used as marker of cell cycle activity.

### Data Availability

The data sets analysed and full sets of results obtained during the current study are available from the corresponding author on reasonable request.

## Electronic supplementary material

Supplementary Information
