# Publisher Correction: Genome-wide linkage and association study implicates the 10q26 region as a major genetic contributor to primary nonsyndromic vesicoureteric reflux

**DOI:** 10.1038/s41598-017-17992-w

**Published:** 2018-01-08

**Authors:** John M. Darlow, Rebecca Darlay, Mark G. Dobson, Aisling Stewart, Pimphen Charoen, Jennifer Southgate, Simon C. Baker, Yaobo Xu, Manuela Hunziker, Heather J. Lambert, Andrew J. Green, Mauro Santibanez-Koref, John A. Sayer, Timothy H. J. Goodship, Prem Puri, Adrian S. Woolf, Rajko B. Kenda, David E. Barton, Heather J. Cordell

**Affiliations:** 1 Department of Clinical Genetics, Our Lady’s Children’s Hospital, Crumlin, Dublin 12 Ireland; 2 National Children’s Research Centre, Our Lady’s Children’s Hospital, Crumlin, Dublin 12 Ireland; 3 Institute of Genetic Medicine, Newcastle University, Central Parkway, Newcastle upon Tyne, NE1 3BZ UK; 4 UCL Institute of Health Informatics, University College, London, NW1 2DA UK; 5 Department of Tropical Hygiene, Faculty of Tropical Medicine, Mahidol University, Bangkok, 10400 Thailand; 6 Jack Birch Unit of Molecular Carcinogenesis, Department of Biology, University of York, York, YO10 5DD UK; 7 University Children’s Hospital Zurich, Steinwiesstrasse 75, 8032 Zurich, Switzerland; 8 Royal Victoria Infirmary, Newcastle upon Tyne, NE1 4LP UK; 9 University College Dublin School of Medicine, Our Lady’s Children’s Hospital, Crumlin, Dublin 12 Ireland; 10 University College Dublin, Stillorgan Rd, Belfield, Dublin 4 Ireland; 11 Division of Cell Matrix Biology and Regenerative Medicine, Faculty of Biology, Medicine and Health, University of Manchester, Manchester, UK; 12 Royal Manchester Children’s Hospital and Manchester Academic Health Sciences Centre, Manchester, UK; 13 Department of Pediatric Nephrology, University Medical Centre Ljubljana, Ljubljana, Slovenia

Correction to: *Scientific Reports* https://doi.org/10.1038/s41598-017-15062-9, published online 03 November 2017

The original HTML version of this Article contained a typographical error in the publication date ‘03 November 2017’ which was incorrectly given as ‘06 November 2017’. This has now been corrected in the HTML version of the Article.

