# Supplementary material for: Genome-wide linkage and association study implicates the 10q26 region as a major genetic contributor to primary nonsyndromic vesicoureteric reflux
Source: Sci Rep. 2017 Nov 3;7:14595. doi: 10.1038/s41598-017-15062-9 (PMC5668427; doi:10.1038/s41598-017-15062-9)
Supplement: Supplementary file 1 — Supplementary Information [file 41598_2017_15062_MOESM1_ESM.pdf]

Genome-wide linkage and association study implicates the 10q26 region as a major genetic contributor to primary nonsyndromic vesicoureteric reflux

John M. Darlow[1,2,+], Rebecca Darlay[3,+], Mark G. Dobson[1,2], Aisling Stewart[3], Pimphen Charoen[4,5], Jennifer Southgate[6], Simon C. Baker[6], Yaobo Xu[3], Manuela Hunziker[2,7], Heather J. Lambert[8], Andrew J. Green[1,9], Mauro Santibanez-Koref[3], John A. Sayer[3], Timothy H.J. Goodship[3], Prem Puri[2,10], Adrian S. Woolf[11,12], Rajko B. Kenda[13], David E. Barton[1,9], and Heather J. Cordell[3,*]

[1]Department of Clinical Genetics, Our Lady's Children's Hospital, Crumlin, Dublin 12, Ireland
[2]National Children's Research Centre, Our Lady's Children's Hospital, Crumlin, Dublin 12, Ireland
[3]Institute of Genetic Medicine, Newcastle University, Central Parkway, Newcastle upon Tyne, NE1 3BZ, UK
[4]UCL Institute of Health Informatics, University College, London, NW1 2DA, UK
[5]Department of Tropical Hygiene, Faculty of Tropical Medicine, Mahidol University, Bangkok 10400, Thailand [6]Jack Birch Unit of Molecular Carcinogenesis, Department of Biology, University of York, York, YO10 5DD, UK [7]University Children's Hospital Zurich, Steinwiesstrasse 75, 8032 Zurich, Switzerland
[8]Royal Victoria Infirmary, Newcastle upon Tyne, NE1 4LP, UK
[9]University College Dublin Department of Medical Genetics, Our Lady's Children's Hospital, Crumlin, Dublin 12, Ireland
[10]University College Dublin, Stillorgan Rd, Belfield, Dublin 4, Ireland
[11]Division of Cell Matrix Biology and Regenerative Medicine, Faculty of Biology, Medicine and Health, University of Manchester, UK
[12]Royal Manchester Children's Hospital and Manchester Academic Health Sciences Centre, Manchester, UK
[13]Department of Pediatric Nephrology, University Medical Centre Ljubljana, Ljubljana, Slovenia
[*]heather.cordell@newcastle.ac.uk
[+]these authors contributed equally to this work

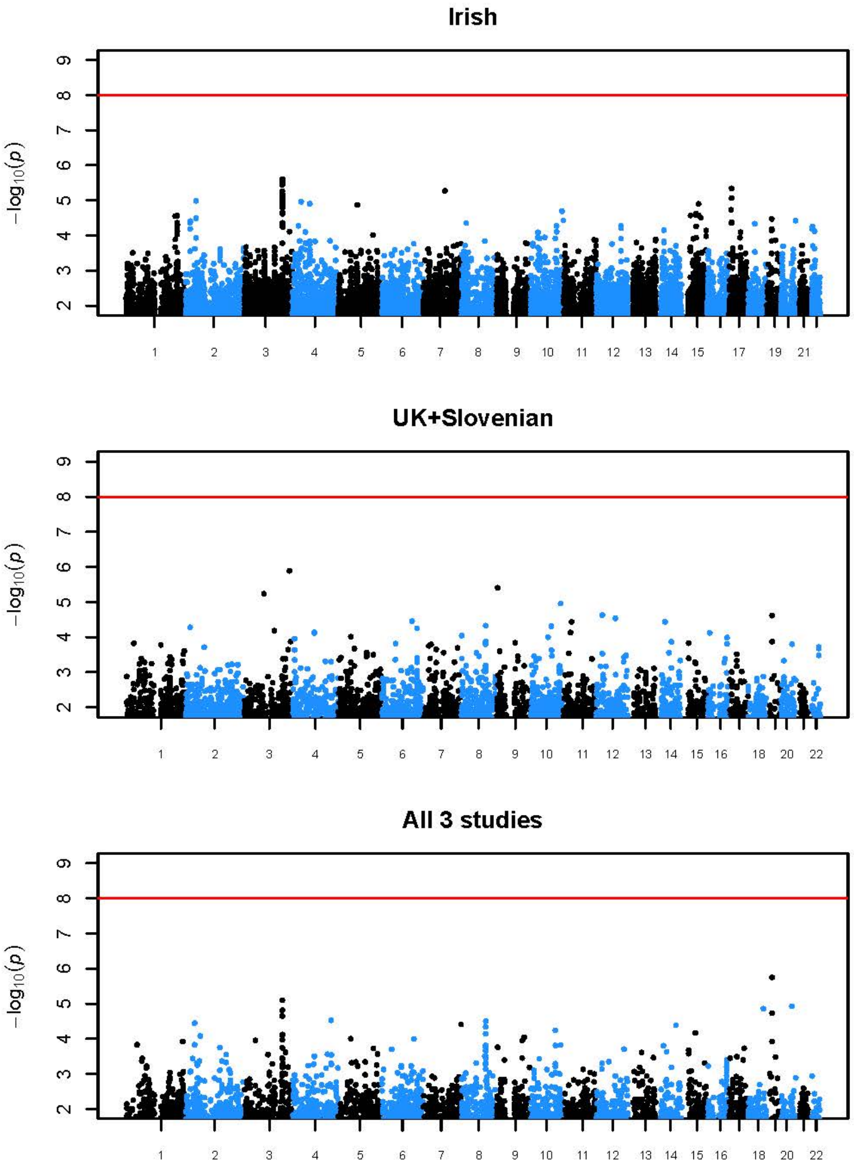

Figure S1: Family-based association (TDT) results at genotyped SNPs. The x axis denotes chromosome (with SNPs ordered by base pair position) while the y axis denotes -log10(p-value). A red horizontal line is drawn at a genome-wide significance threshold of $P=1x10^{-8}$.

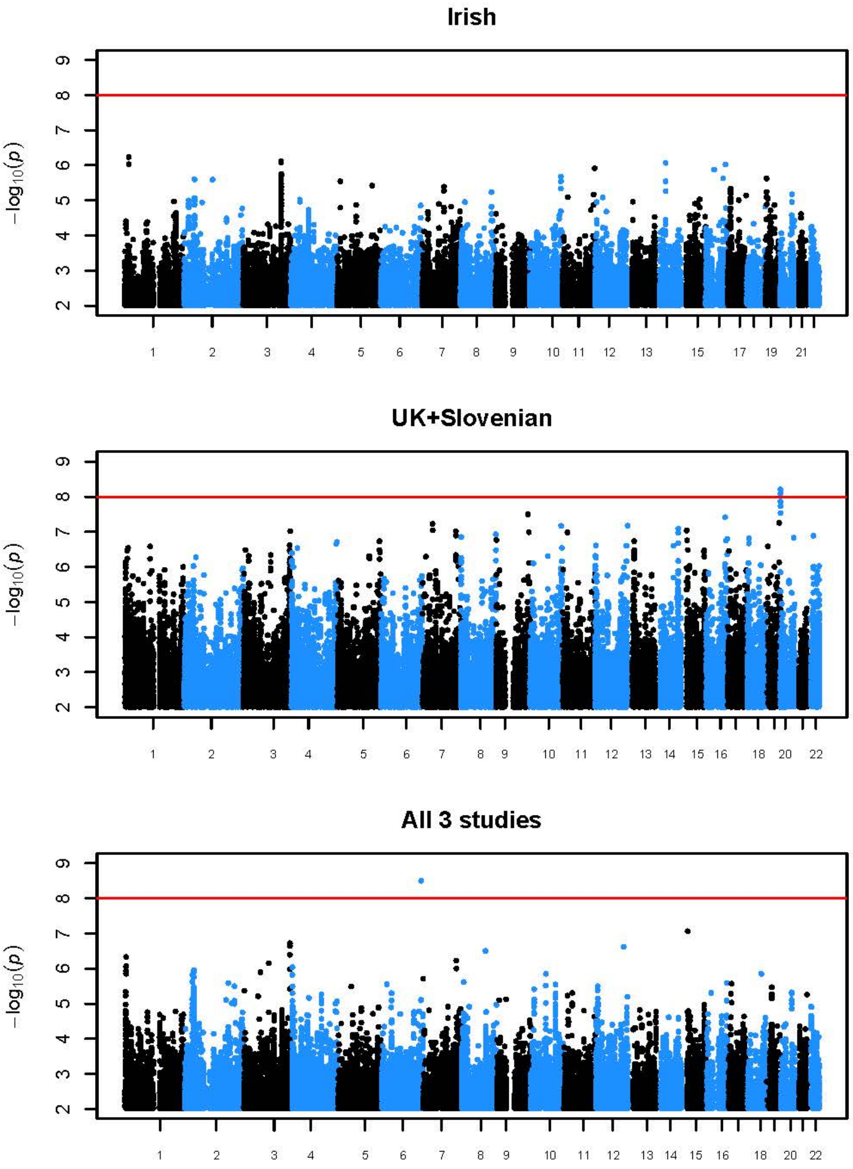

Figure S2: Family-based association (TDT) results at imputed SNPs. The x axis denotes chromosome (with SNPs ordered by base pair position) while the y axis denotes -log10(p-value). A red horizontal line is drawn at a genome-wide significance threshold of *P*=1x10-8.

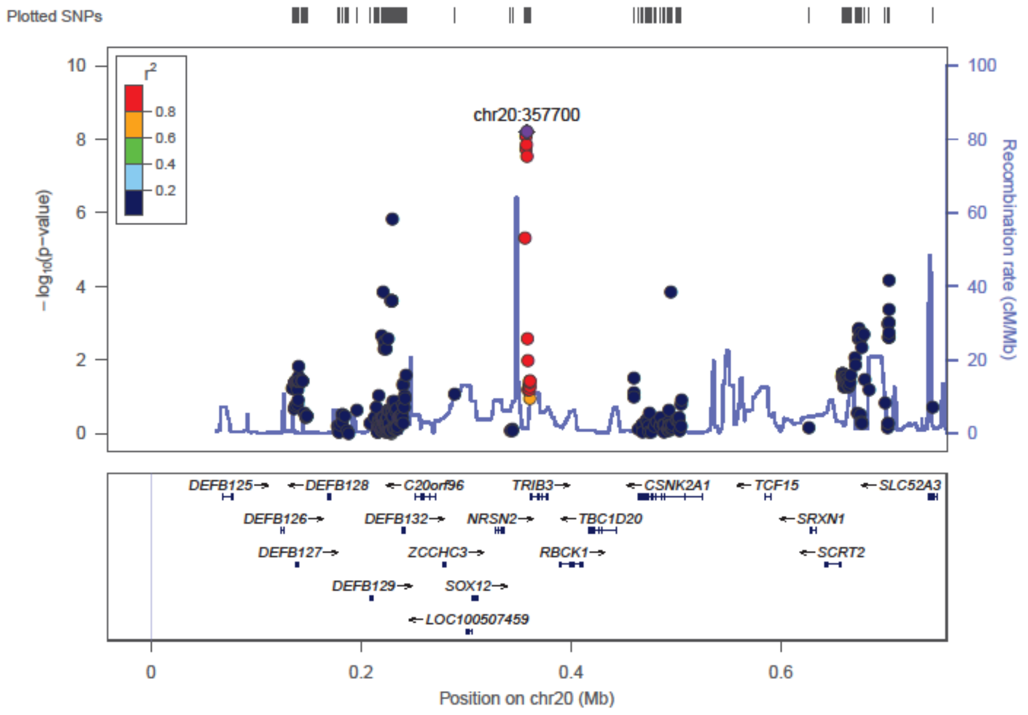

Figure S3: LocusZoom plot (GRCh37 positions) of TDT results at imputed SNPs in the UK/Slovenian cohorts, centered around index SNP rs6138998 (chr20:357700) on chromosome 20. The index SNP is shown as a purple diamond, while other SNPs are colored according to their correlation with the index SNP.

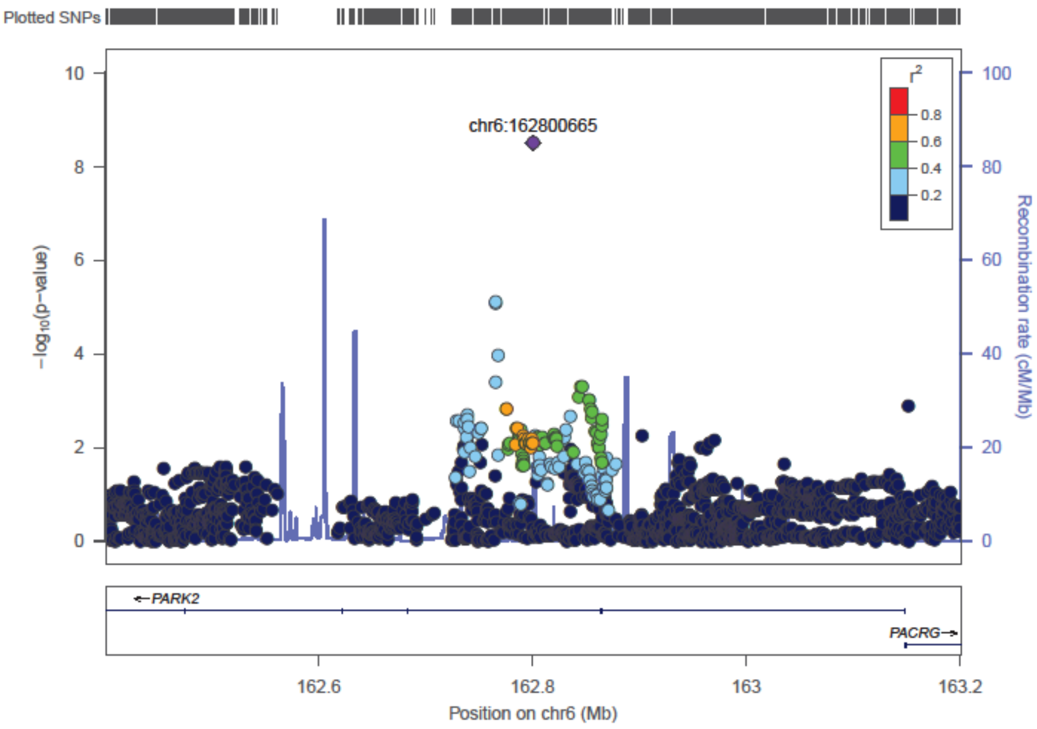

Figure S4: LocusZoom plot (GRCh37 positions) of TDT results at imputed SNPs in the combined Irish/UK/Slovenian cohorts, centered around index SNP rs11759064 (chr6:162800665) on chromosome 6. The index SNP is shown as a purple diamond, while other SNPs are colored according to their correlation with the index SNP.

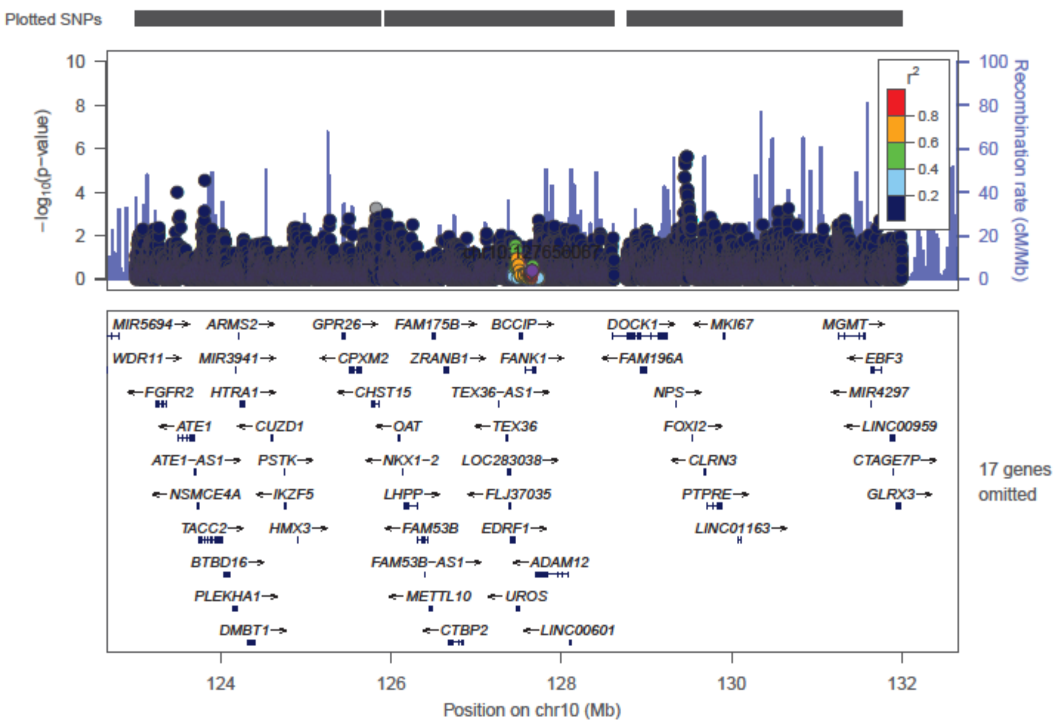

Figure S5: LocusZoom plot (GRCh37 positions) of TDT results at imputed SNPs in the Irish cohort, centered around index SNP rs7907300 (chr10:127656067) on chromosome 10. The index SNP is shown as a purple diamond, while other SNPs are colored according to their correlation with the index SNP.

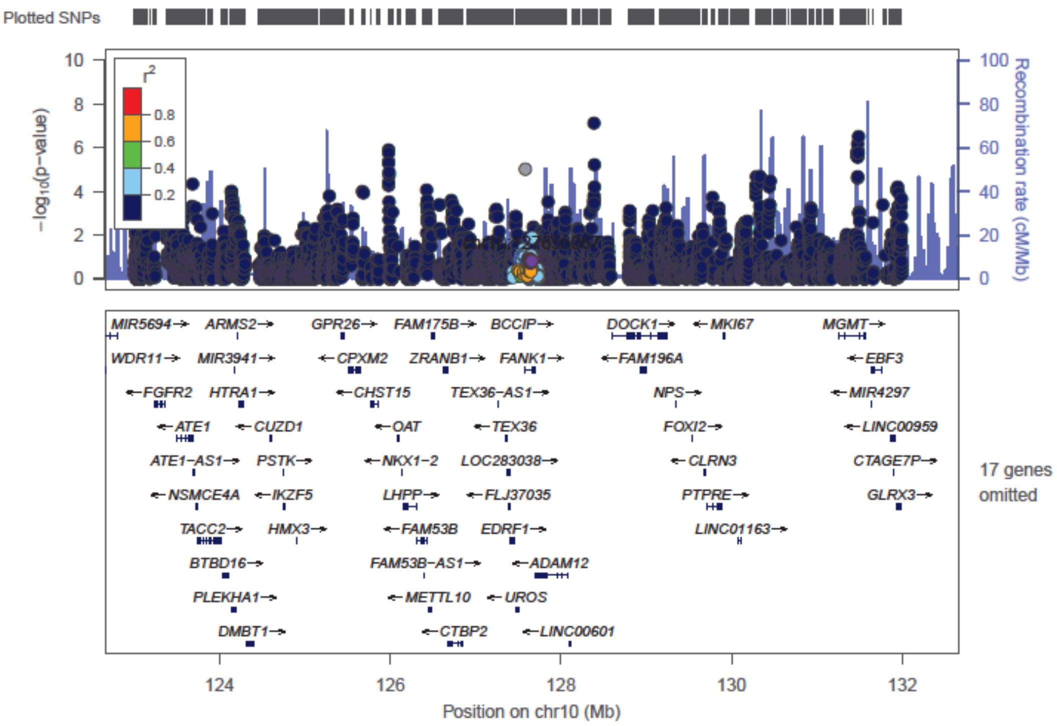

Figure S6: LocusZoom plot (GRCh37 positions) of TDT results at imputed SNPs in the UK/Slovenian cohorts, centered around index SNP rs7907300 (chr10:127656067) on chromosome 10. The index SNP is shown as a purple diamond, while other SNPs are colored according to their correlation with the index SNP.

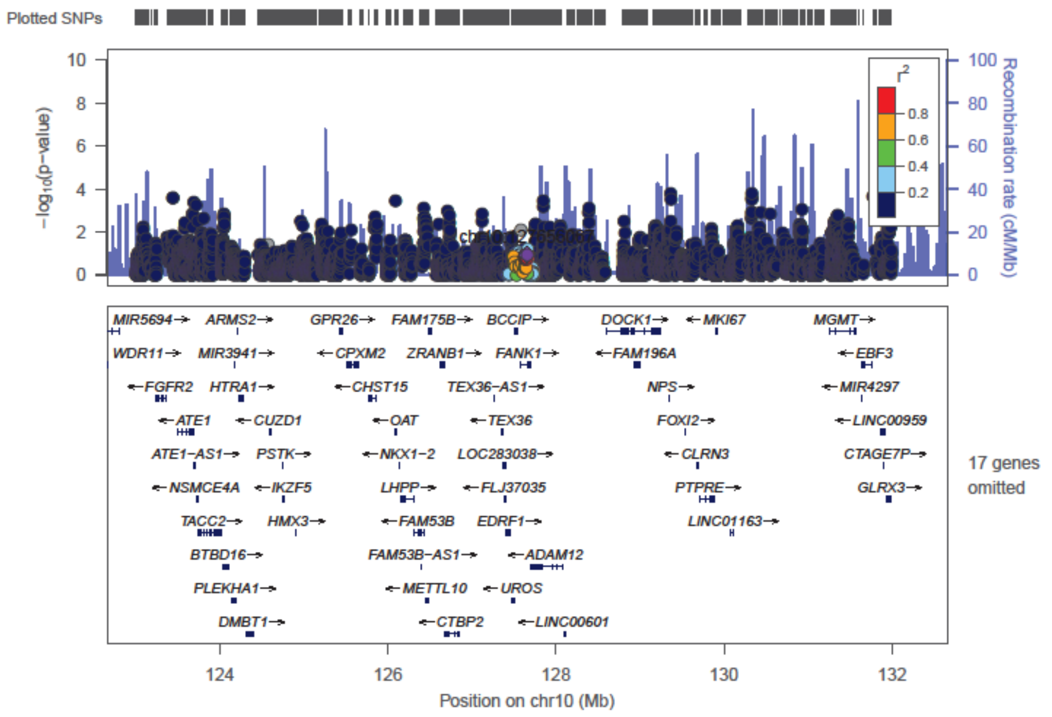

Figure S7: LocusZoom plot (GRCh37 positions) of TDT results at imputed SNPs in the combined Irish/UK/Slovenian cohorts, centered around index SNP rs7907300 (chr10:127656067) on chromosome 10. The index SNP is shown as a purple diamond, while other SNPs are colored according to their correlation with the index SNP.

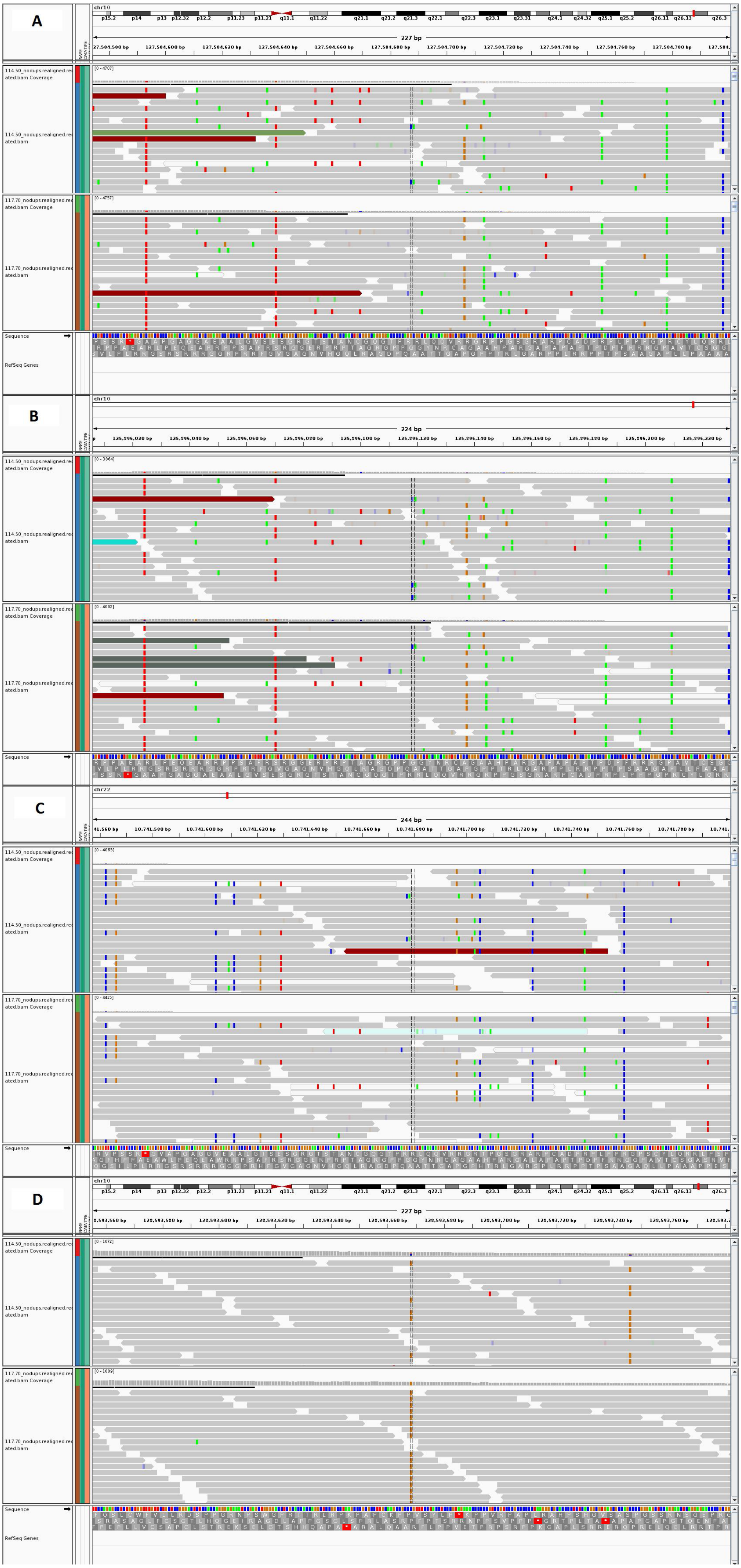
A
chr10
227 bp
114.50_nodups.realigned.bam Coverage
114.50_nodups.realigned.bam
117.70_nodups.realigned.bam Coverage
117.70_nodups.realigned.bam
Sequence
RefSeq Genes
B
chr10
224 bp
C
chr22
244 bp
D
chr10
227 bp

Figure S8: Visual representation of .bam files in IGV for a 227 bp window of the same aligned reads mapped to different genome builds and loci. Samples 114.50 and 117.30 are representative of the cohort. Bases matching the reference are coloured grey, variant bases are coloured: green = A, blue = C, orange = G, red = T. Coloured reads are coloured by insert size according to the default IGV scheme, as an indicator of the quality of the alignment: red = inferred insert size is larger than expected and indicative of possible deletions, blue = inferred insert size is smaller than expected and indicative of possible insertions, green = paired end read mate is on chromosome 3, dark grey = paired end read mate is on chromosome 12. A) Alignment of reads mapped to Chr10:127584574-127584800 on GRCh37 (hg19) reference genome. B) Alignment of reads mapped to Chr10:125896006-125896229 on the GRCh38 reference genome. C) Alignments of reads mapped to Chr22:10741557-10741800 on the GRCh38 reference genome. D) Reads mapped to Chr10:128593555-128593781 on GRCh37 to illustrate a 'normal' alignment, in order to show how comparatively few variants one would usually find in a window of this size. The G/C SNV seen is a common SNP, rs12781269, in the promoter flanking region of DOCK1.

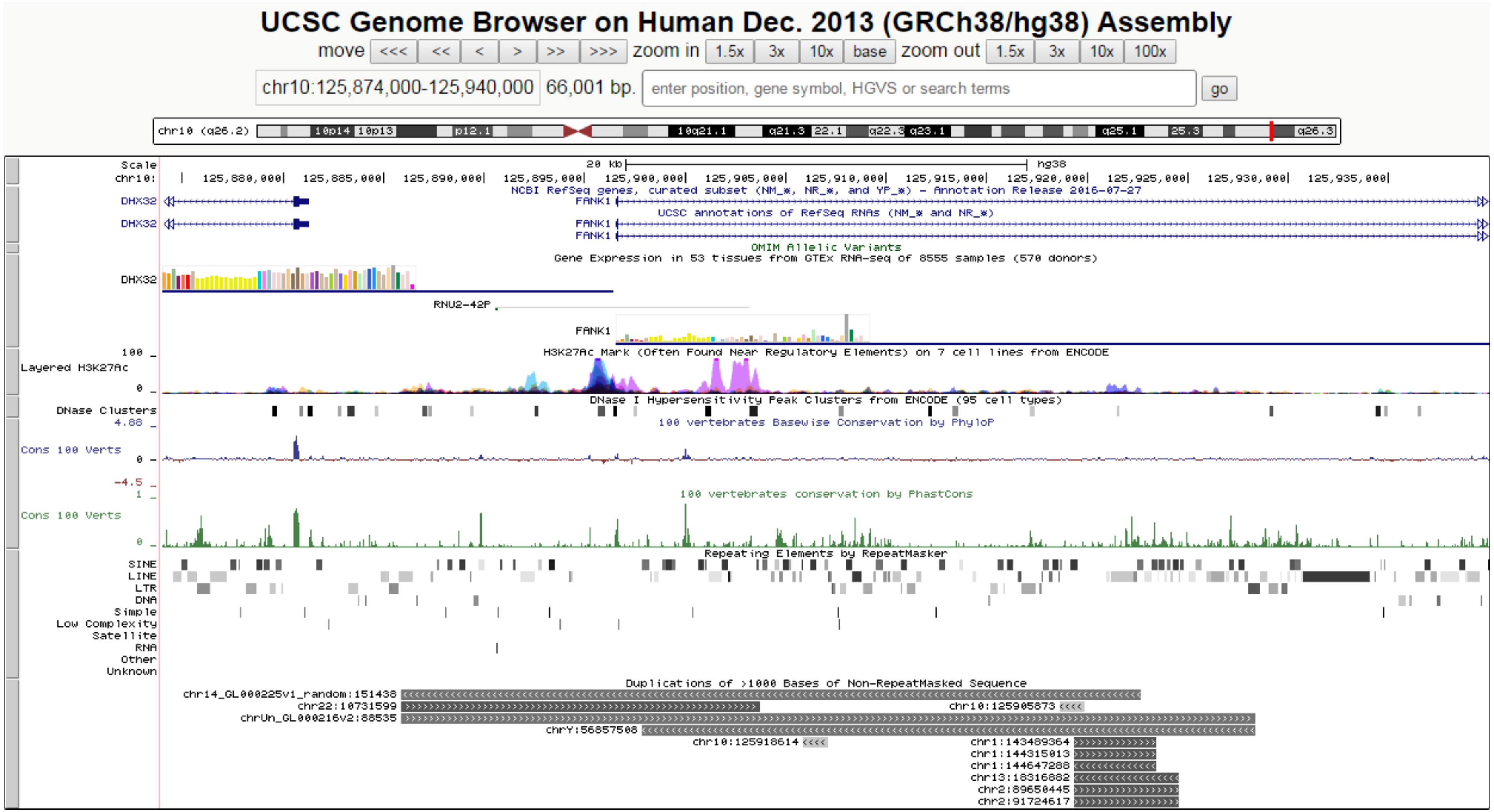

Figure S9: Segmental duplication of a 42.4 kb region of chromosome 10 from 125885884 to 125928375 (GRCh38 assembly), as shown by UCSC Genome Browser

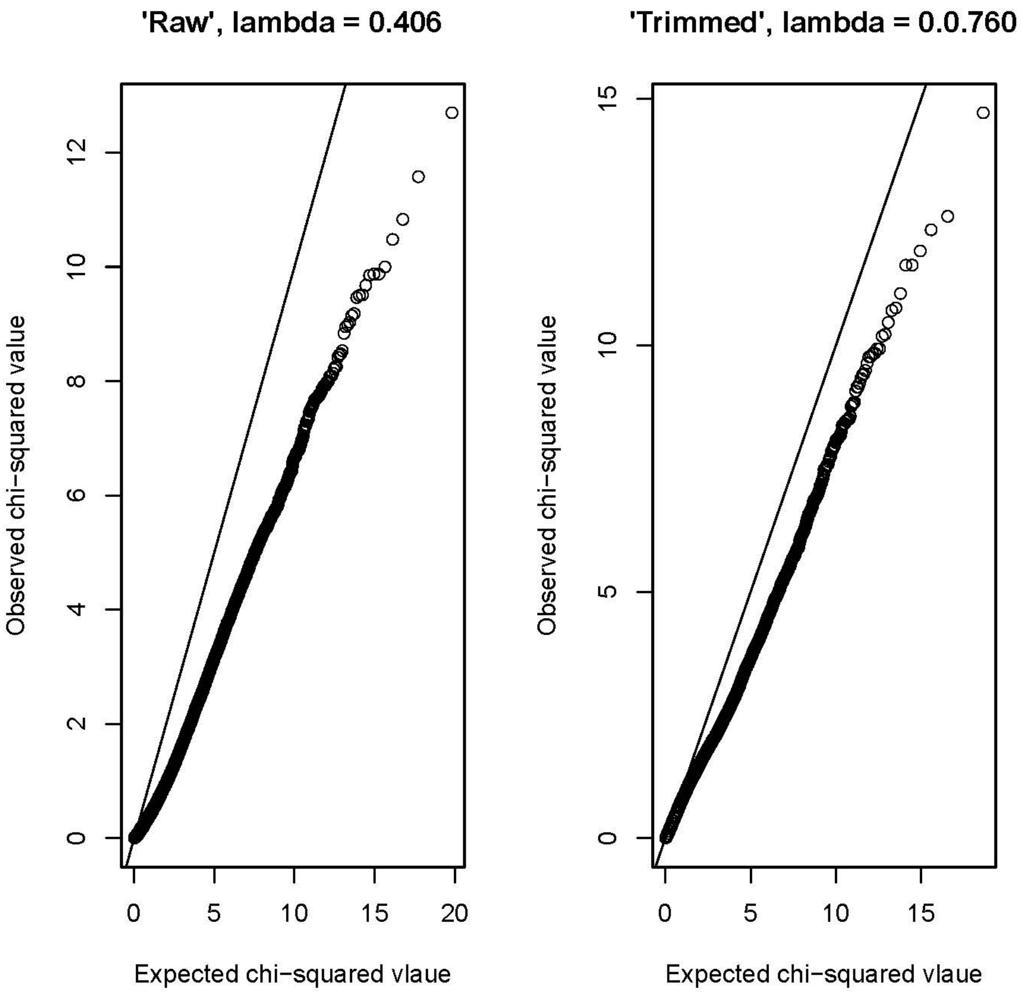

Figure S10: Quantile-Quantile (Q-Q) plots showing the ordered -log10(p-values) from TASER against their expected values under the null hypothesis of no differences between cases and controls. Left hand plot shows the 'raw' results where every allele seen in any sequencing read is counted. Right hand plot shows the 'trimmed' results where only alleles seen at least 5 times in that position in the cases (high read depth) and at least twice in the controls (low read depth) are counted.

SIGNAL	COHORT	CHR	SNP	RSID	BP	A1	A2	MAF	T	U	OR	CHISQ	P
1	Irish	20	20:356785	rs6037460	356785	A	G	0.3329	183	165	1.109	0.931	0.3346
1	Irish	20	20:356861	rs6051546	356861	G	A	0.3346	183	163	1.123	1.156	0.2823
1	Irish	20	20:357050	rs6084242	357050	C	T	0.3329	183	165	1.109	0.931	0.3346
1	Irish	20	20:357128	rs6051547	357128	A	T	0.3313	180	164	1.098	0.744	0.3883
1	Irish	20	20:357700	rs6138998	357700	T	C	0.3321	185	163	1.135	1.391	0.2383
1	UK/Slovenian	20	20:356785	rs6037460	356785	A	G	0.2168	42	114	0.3684	33.23	8.19E-09
1	UK/Slovenian	20	20:356861	rs6051546	356861	G	A	0.2168	42	114	0.3684	33.23	8.19E-09
1	UK/Slovenian	20	20:357050	rs6084242	357050	C	T	0.2175	46	118	0.3898	31.61	1.89E-08
1	UK/Slovenian	20	20:357128	rs6051547	357128	A	T	0.2203	48	122	0.3934	32.21	1.38E-08
1	UK/Slovenian	20	20:357700	rs6138998	357700	T	C	0.232	53	132	0.4015	33.74	6.32E-09
1	Combined Irish/UK/Slovenian	20	20:356785	rs6037460	356785	A	G	0.2752	225	279	0.8065	5.786	0.01616
1	Combined Irish/UK/Slovenian	20	20:356861	rs6051546	356861	G	A	0.2759	225	277	0.8123	5.386	0.02029
1	Combined Irish/UK/Slovenian	20	20:357050	rs6084242	357050	C	T	0.2755	229	283	0.8092	5.695	0.01701
1	Combined Irish/UK/Slovenian	20	20:357128	rs6051547	357128	A	T	0.2757	228	286	0.7972	6.545	0.01052
1	Combined Irish/UK/Slovenian	20	20:357700	rs6138998	357700	T	C	0.2812	238	295	0.8068	6.096	0.01355
2	Irish	6	6:162800665	rs11759064	162800665	T	C	0.1264	38	80	0.475	14.95	0.00011
2	UK/Slovenian	6	6:162800665	rs11759064	162800665	T	C	0.1323	41	93	0.4409	20.18	7.05E-06
2	Combined Irish/UK/Slovenian	6	6:162800665	rs11759064	162800665	T	C	0.1298	79	173	0.4566	35.06	3.19E-09

Table S1: TDT results from imputed SNPs passing significance threshold P<2E-08 in individual or combined cohorts. A1 refers to the minor allele, and T and U to the number of times it is transmitted or untransmitted from heterozygous parents to affected offspring.

					Closest linkage score	
**Gene**	**Start**	**End**	**Orientation**	**Notes**	**SNP**	**HLOD**
WDR11	122610687	122669036	+	WD repeat domain 11	rs2420779	0.4842
FGFR2	123237848	123359792	-	Fibroblast growth factor receptor 2	rs1693682	0.6330
ATE1	123499939	123688316	-	Arginyltransferase 1	rs12354464	0.6848
ATE1-AS1	123687827	123711480	+	arginyltransferase 1 antisense1	rs12354464	0.6848
NSMCE4A	123716603	123734732	-	NSE4 homolog A. SMC5-SMC6 complex component	rs12354464	0.6848
TACC2	123748689	124014060	+	transforming acidic coiled-coil containing protein 2	rs2461215	0.8433
BTBD16	124030821	124097677	+	BTB domain containing 16	rs7088058	0.9436
PLEKHA1	124134212	124191867	+	pleckstrin homology domain containing A1	rs7088058	0.9436
MIR3941	124176481	124176583	+	microRNA 3941; post-transcriptional regulation of gene expression	rs7088058	0.9436
ARMS2	124214169	124216868	+	age-related maculopathy susceptibility 2	rs7088058	0.9436
HTRA1	124221040	124274424	+	HtrA serine peptidase 1	rs7088058	0.9436
DMBT1	124320181	124403252	+	deleted in malignant brain tumours 1	rs17104231	1.1216
C10orf120	124457225	124459338	-	Chromosome 10 open reading frame 120	rs17104231	1.1216
DMBT1P1	124516210	124558696	+	deleted in malignant brain tumours pseudogene 1	rs17104231	1.1216
FAM24B-CUZD1	NA	NA	-	NA	NA	NA
CUZD1	124591665	124639146	-	CUB and zona pellucida like domains 1	rs17104231	1.1216
FAM24B	124608594	124639157	-	family with sequence similarity 24 member B	rs17104231	1.1216
LOC399815	124643295	124658230	+	Chromosome 10 open reading frame 88 pseudogene	rs17104231	1.1216
FAM24A	124670217	124672627	+	family with sequence similarity 24 member A	rs17104231	1.1216
C10orf88[a]	124690419	124713919	-	Chromosome 10 open reading frame 88	rs17104231	1.1216
PSTK	124713897	124757029	+	phosphoseryl-tRNA kinase	rs17104231	1.1216

IKZF5	124750322	124768333	-	IKAROS family zinc finger 5; transcription factor	rs17104231	1.1216
ACADSB[a]	124768495	124817827	+	acyl-CoA dehydrogenase, short/branched chain	rs17104231	1.1216
HMX3	124895478	124897257	+	H6 family homeobox 3; transcription factor	rs7918922	1.3690
HMX2[a]	124907638	124910188	+	H6 family homeobox 2; transcription factor	rs7918922	1.3690
BUB3[a]	124913793	124924886	+	mitotic check point protein	rs7918922	1.3690
GPR26	125415871	125454123	+	G protein-coupled receptor 26	rs2479901	1.6074
CPXM2	125465723	125699783	-	carboxypetidase X, M14 family member 1	rs17680424	2.6678
CHST15	125767184	125853206	-	carbohydrate sulfotransferase 15	rs17680424	2.6678
OAT	126085872	126107545	-	ornithine aminotransferase	rs4962728	2.8835
NKX1-2	126135592	126138753	-	NK1 homeobox 2	rs4962728	2.8835
LHPP	126150403	126306457	+	phospholysine phosphohistidine inorganic pyrophosphate phosphatase	rs11245344	4.0428
FAM53B	126307861	126432838	-	family with sequence similarity 53 member B	rs11245344	4.0428
FAM53B-AS1	126392194	126402786	+	family with sequence similarity 53 member B antisense 1	rs11245344	4.0428
METTL10	126436718	126480439	-	lysine methyltransferase 2	rs11245344	4.0428
FAM175B	126490354	126525239	+	ABRAXAS2; BRISC complex subunit	rs11245344	4.0428
ZRANB1	126630692	126676758	+	zinc finger RANBP2-type containing 1	rs11245344	4.0428
CTBP2	126676421	126849739	-	C-terminal binding protein 2; transcriptional repressor	rs11245344	4.0428
MIR4296	126721352	126721439	-	microRNA 4296; post-transcriptional regulation of gene expression	rs11244392	4.3156
TEX36-AS1	127262940	127267014	+	testis expressed 36 antisense 1	rs2365818	4.6879
TEX36	127265091	127371713	-	testis expressed 36	rs2365818	4.6879
LOC283038	127371812	127398627	+	uncharacterised	rs1872006	4.8556
FLJ37035	127389005	127408135	-	uncharacterised	rs1872006	4.8556
EDRF1	127408084	127452712	+	erthyroid differentiation regulatory factor 1; inhibits binding of transcription factor GATA1	rs1872006	4.8556

EDRF1-AS1	127433296	127440679	-	erthyroid differentiation regulatory factor 1 antisense 1	rs1872006	4.8556
MMP21	127455022	127464390	-	matrix metallopeptidase 21	rs1872006	4.8556
UROS	127477146	127511817	-	uroporphyrinogen III synthase	rs7907300	4.9066
MIR4484	127508309	127508391	+	microRNA 4484; post-transcriptional regulation of gene expression	rs7907300	4.9066
BCCIP	127512115	127542264	+	BRCA2 and CDKN1A interacting protein	rs7907300	4.9066
DHX32	127524906	127585005	-	DEAH-box helicase 32	rs7907300	4.9066
FANK1	127585108	127698161	+	fibronectin type III and ankyrin repeat domains 1	rs7907300	4.9066
FANK1-AS1	127660757	127661695	-	fibronectin type III and ankyrin repeat domains 1 antisense 1	rs7907300	4.9066
ADAM12	127700950	128077024	-	ADAM metallopeptidase 12	rs10794060	4.7134
LINC00601	128102438	128110448	-	long intergenic non-protein coding RNA 601	rs7084896	4.4969
C10orf90	128113566	128359079	-	Chromosome 10 open reading frame 90	rs12246110	4.1524
DOCK1	128593978	129250781	+	dedicator of cytokinese 1	rs12415496	3.7981
FAM196A	128933694	128994422	-	family with sequence similarity 196 member A	rs4622171	3.3823
NPS	129347613	129350935	+	neuropeptide S	rs3740547	3.5271
FOXI2	129535499	129539450	+	forkhead box I2	rs4751461	3.5861
CLRN3	129676105	129691211	-	clarin 3	rs2148895	3.3913
PTPRE	129705325	129884119	+	protein tyrosine phosphatase receptor type E	rs2148895	3.3913
MKI67	129894923	129924649	-	marker of proliferation Ki-67	rs2148895	3.3913
LINC01163	130084213	130115990	+	long intergenic non-protein coding RNA 1163	rs7900125	3.3749
MGMT	131265448	131566271	+	O-6-methylguanine-DNA methyltranserase	rs11016797	2.0239
EBF3	131633547	131762105	-	early B cell factor 3; DNA binding transcription factor	rs3793907	1.9411
MIR4297	131641563	131641638	-	microRNA 4297; post-transcriptional regulation of gene expression	rs3793907	1.9411
LINC00959	131864638	131909081	-	long intergenic non-protein coding RNA 959	rs1183343	1.4009

CTAGE7P	131904273	131907095	+	CTAGE family member 7, pseudogene	rs1183343	1.4009
GLRX3	131934663	131982785	+	glutaredoxin	rs318934	1.4381

Table S2: Genes in the 9Mb targeted region of chromosome 10. Start and End bp positions are aligned to GRCh37 (Feb 2014).

[a] these genes are omitted from Figure 3

SIGNAL	COHORT	CHR	SNP	RSID	BP	A1	A2	MAF	T	U	OR	CHISQ	P
1	Irish	10	10:129452307	rs2386898	129452307	C	T	0.1476	147	77	1.909	21.88	2.91E-06
1	Irish	10	10:129455999	rs11018245	129455999	G	A	0.1481	148	79	1.873	20.97	4.66E-06
1	Irish	10	10:129456705	rs11018246	129456705	G	A	0.1481	148	79	1.873	20.97	4.66E-06
1	Irish	10	10:129469918	rs60182575	129469918	G	A	0.1468	144	74	1.946	22.48	2.13E-06
1	Irish	10	10:129474049	rs12247046	129474049	C	G	0.1468	144	74	1.946	22.48	2.13E-06
1	Irish	10	10:129474336	rs60100674	129474336	G	T	0.1468	144	74	1.946	22.48	2.13E-06
1	Irish	10	10:129474758	rs78697163	129474758	G	C	0.1468	144	74	1.946	22.48	2.13E-06
1	UK/Slovenian	10	10:129452307	rs2386898	129452307	C	T	0.1281	22	43	0.5116	6.785	0.009195
1	UK/Slovenian	10	10:129455999	rs11018245	129455999	G	A	0.1267	22	44	0.5	7.333	0.006769
1	UK/Slovenian	10	10:129456705	rs11018246	129456705	G	A	0.1267	22	44	0.5	7.333	0.006769
1	UK/Slovenian	10	10:129469918	rs60182575	129469918	G	A	0.1271	24	43	0.5581	5.388	0.02028
1	UK/Slovenian	10	10:129474049	rs12247046	129474049	C	G	0.1247	24	43	0.5581	5.388	0.02028
1	UK/Slovenian	10	10:129474336	rs60100674	129474336	G	T	0.1261	23	39	0.5897	4.129	0.04215
1	UK/Slovenian	10	10:129474758	rs78697163	129474758	G	C	0.1267	24	40	0.6	4	0.0455
1	Combined Irish/UK/Slovenian	10	10:129452307	rs2386898	129452307	C	T	0.1385	169	120	1.408	8.308	0.003947
1	Combined Irish/UK/Slovenian	10	10:129455999	rs11018245	129455999	G	A	0.138	170	123	1.382	7.539	0.006037
1	Combined Irish/UK/Slovenian	10	10:129456705	rs11018246	129456705	G	A	0.138	170	123	1.382	7.539	0.006037
1	Combined Irish/UK/Slovenian	10	10:129469918	rs60182575	129469918	G	A	0.1374	168	117	1.436	9.126	0.00252
1	Combined Irish/UK/Slovenian	10	10:129474049	rs12247046	129474049	C	G	0.1362	168	117	1.436	9.126	0.00252
1	Combined Irish/UK/Slovenian	10	10:129474336	rs60100674	129474336	G	T	0.137	167	113	1.478	10.41	0.00125
1	Combined Irish/UK/Slovenian	10	10:129474758	rs78697163	129474758	G	C	0.1373	168	114	1.474	10.34	0.001301
2	Irish	10	10:125978765	rs12414492	125978765	A	G	0.3358	194	154	1.26	4.598	0.03201
2	Irish	10	10:125979306	rs12249299	125979306	C	T	0.3354	194	152	1.276	5.098	0.02395

2	Irish	10	10:125979312	rs12244678	125979312	T	C	0.3354	194	152	1.276	5.098	0.02395
2	Irish	10	10:125981562	rs10901829	125981562	T	C	0.335	195	152	1.283	5.329	0.02098
2	UK/Slovenian	10	10:125978765	rs12414492	125978765	A	G	0.2243	42	96	0.4375	21.13	4.29E-06
2	UK/Slovenian	10	10:125979306	rs12249299	125979306	C	T	0.2247	44	103	0.4272	23.68	1.14E-06
2	UK/Slovenian	10	10:125979312	rs12244678	125979312	T	C	0.2227	44	102	0.4314	23.04	1.59E-06
2	UK/Slovenian	10	10:125981562	rs10901829	125981562	T	C	0.2686	88	160	0.55	20.9	4.83E-06
2	Combined Irish/UK/Slovenian	10	10:125978765	rs12414492	125978765	A	G	0.2785	236	250	0.944	0.4033	0.5254
2	Combined Irish/UK/Slovenian	10	10:125979306	rs12249299	125979306	C	T	0.278	238	255	0.9333	0.5862	0.4439
2	Combined Irish/UK/Slovenian	10	10:125979312	rs12244678	125979312	T	C	0.2772	238	254	0.937	0.5203	0.4707
2	Combined Irish/UK/Slovenian	10	10:125981562	rs10901829	125981562	T	C	0.2983	283	312	0.9071	1.413	0.2345
3	Irish	10	10:127580771	rs10901461	127580771	G	A	0.4342	195	202	0.9653	0.1234	0.7253
3	UK/Slovenian	10	10:127580771	rs10901461	127580771	G	A	0.3104	49	104	0.4712	19.77	8.73E-06
3	Combined Irish/UK/Slovenian	10	10:127580771	rs10901461	127580771	G	A	0.3731	244	306	0.7974	6.989	0.008201
4	Irish	10	10:128386150	rs10901656	128386150	C	T	0.471	211	208	1.014	0.02148	0.8835
4	Irish	10	10:128390819	rs10901663	128390819	T	G	0.3108	162	162	1	0	1
4	UK/Slovenian	10	10:128386150	rs10901656	128386150	C	T	0.4094	89	177	0.5028	29.11	6.83E-08
4	UK/Slovenian	10	10:128390819	rs10901663	128390819	T	G	0.2879	79	147	0.5374	20.46	6.09E-06
4	Combined Irish/UK/Slovenian	10	10:128386150	rs10901656	128386150	C	T	0.4384	300	385	0.7792	10.55	0.001163
4	Combined Irish/UK/Slovenian	10	10:128390819	rs10901663	128390819	T	G	0.2986	241	309	0.7799	8.407	0.003737

5	Irish	10	10:131471398	rs11016864	131471398	A	G	0.13	90	94	0.9574	0.08696	0.7681
5	Irish	10	10:131471611	rs11016865	131471611	T	C	0.1309	90	95	0.9474	0.1351	0.7132
5	Irish	10	10:131471968	rs11016866	131471968	A	G	0.1312	89	94	0.9468	0.1366	0.7117
5	Irish	10	10:131472040	rs57547298	131472040	G	T	0.1309	90	95	0.9474	0.1351	0.7132
5	Irish	10	10:131476611	rs56880178	131476611	A	G	0.1312	94	96	0.9792	0.02105	0.8846
5	Irish	10	10:131476971	rs56800605	131476971	T	C	0.1312	94	96	0.9792	0.02105	0.8846
5	Irish	10	10:131477693	rs12255679	131477693	A	G	0.1312	94	96	0.9792	0.02105	0.8846
5	Irish	10	10:131479653	rs68109323	131479653	G	A	0.1324	96	96	1	0	1
5	Irish	10	10:131479665	rs67372222	131479665	G	A	0.1312	94	96	0.9792	0.02105	0.8846
5	Irish	10	10:131479680	rs12245575	131479680	T	C	0.1312	94	96	0.9792	0.02105	0.8846
5	Irish	10	10:131489398	rs11016878	131489398	A	G	0.119	85	83	1.024	0.02381	0.8774
5	UK/Slovenian	10	10:131471398	rs11016864	131471398	A	G	0.07782	16	57	0.2807	23.03	1.60E-06
5	UK/Slovenian	10	10:131471611	rs11016865	131471611	T	C	0.07782	16	57	0.2807	23.03	1.60E-06
5	UK/Slovenian	10	10:131471968	rs11016866	131471968	A	G	0.07819	17	58	0.2931	22.41	2.20E-06
5	UK/Slovenian	10	10:131472040	rs57547298	131472040	G	T	0.07819	17	58	0.2931	22.41	2.20E-06
5	UK/Slovenian	10	10:131476611	rs56880178	131476611	A	G	0.08111	19	63	0.3016	23.61	1.18E-06
5	UK/Slovenian	10	10:131476971	rs56800605	131476971	T	C	0.08031	19	61	0.3115	22.05	2.66E-06
5	UK/Slovenian	10	10:131477693	rs12255679	131477693	A	G	0.08254	20	66	0.303	24.6	7.04E-07
5	UK/Slovenian	10	10:131479653	rs68109323	131479653	G	A	0.08491	23	69	0.3333	23	1.62E-06
5	UK/Slovenian	10	10:131479665	rs67372222	131479665	G	A	0.08349	23	69	0.3333	23	1.62E-06
5	UK/Slovenian	10	10:131479680	rs12245575	131479680	T	C	0.08427	23	69	0.3333	23	1.62E-06
5	UK/Slovenian	10	10:131489398	rs11016878	131489398	A	G	0.08004	16	61	0.2623	26.3	2.93E-07
5	Combined Irish/UK/Slovenian	10	10:131471398	rs11016864	131471398	A	G	0.1007	106	151	0.702	7.879	0.005
5	Combined Irish/UK/Slovenian	10	10:131471611	rs11016865	131471611	T	C	0.1011	106	152	0.6974	8.202	0.004185
5	Combined Irish/UK/Slovenian	10	10:131471968	rs11016866	131471968	A	G	0.1013	106	152	0.6974	8.202	0.004185
5	Combined Irish/UK/Slovenian	10	10:131472040	rs57547298	131472040	G	T	0.1012	107	153	0.6993	8.138	0.004334
5	Combined Irish/UK/Slovenian	10	10:131476611	rs56880178	131476611	A	G	0.1029	113	159	0.7107	7.779	0.005285

5	Combined Irish/UK/Slovenian	10	10:131476971	rs56800605	131476971	T	C	0.1025	113	157	0.7197	7.17	0.007412
5	Combined Irish/UK/Slovenian	10	10:131477693	rs12255679	131477693	A	G	0.1037	114	162	0.7037	8.348	0.003861
5	Combined Irish/UK/Slovenian	10	10:131479653	rs68109323	131479653	G	A	0.1055	119	165	0.7212	7.451	0.006341
5	Combined Irish/UK/Slovenian	10	10:131479665	rs67372222	131479665	G	A	0.1041	117	165	0.7091	8.17	0.004258
5	Combined Irish/UK/Slovenian	10	10:131479680	rs12245575	131479680	T	C	0.1045	117	165	0.7091	8.17	0.004258
5	Combined Irish/UK/Slovenian	10	10:131489398	rs11016878	131489398	A	G	0.09711	101	144	0.7014	7.547	0.006011

Table S3: TDT results from imputed SNPs in the 10q26 region passing significance threshold P<1E-05 in individual or combined cohorts. A1 refers to the minor allele, and T and U to the number of times it is transmitted or untransmitted from heterozygous parents to affected offspring.

Gene	Exonic variants	N predicted deleterious	N families
FANK1	5	2	3
CTBP2	6	0 (plus an additional 5 discounted as within segmental duplication)	6
MKI67	6	2 (plus an additional 1 discounted as within segmental duplication)	6
TACC2	2	0	2
DMBT1	2	2	3
ZRANB1	1	0	4
CLRN3	1	0	2

Table S4: Counts of rare variants (AAF < 0.05 in 1000GP/ESP) found in exome sequence of genes in the 10q26 region. Whole exome sequencing was carried out in 29 individuals from 8 families.

Position (GRCh37)	Variant (REF/ALT)	rsID	AAF (CEU)	Amino Acid change	family	SIFT score	Prediction
127668751	C/T	rs17153879	0.005	P>L	SLOV_028	0.03	Deleterious
127668863	GAA/-	rs146106149	0.025	E deletion	DUB_15	-	In-frame deletion
127693479	A/G	rs41302923	0.015	H>R	SLOV_028	0.02	Deleterious
127697001	G/A	rs139642438	0.001 (ESP6500)	G>E	SLOV_121	0.32	Tolerated
127697997	G/T	rs17153976	0.005	C>F	SLOV_028	0.45	Tolerated

Table S5: Exome (coding) variants found in *FANK1* in 3 families from whole exome sequencing. All are consistent with IBD sharing within each family.

Position (GRCh37)	Variant (REF/ALT)	rsID	Gene	N individuals	AAF in VUR cases
124133947	G/C	rs72830779	PLEKHA1	2	0.03125
124220567	G/A	rs148152439	HTRA1	2	0.03125
126138911	G/A	rs140024768	NKX1-2	2	0.03125
129691655	A/G	rs182013372	CLRN3	2	0.03125
129924568	T/A	rs151328961	MKI67	3	0.09375
131934190	T/C	rs11017099	GLRX3	8	0.125
131934191	A/G	rs11017100	GLRX3	8	0.125
131934201	A/G	rs11017101	GLRX3	8	0.125
131934203	C/T	-	GLRX3	8	0.125
131934207	T/G	-	GLRX3	8	0.125
131934212	T/C	rs11017102	GLRX3	8	0.125

Table S6: Rare variants (AAF<0.01) shared in 2 or more individuals, found in the promoter region of genes other than *FANK1*, from targeted sequencing of 10q26.

Position (GRCh37)	REF	ALT	VarScan Allele counts	GATK re-call Allele counts
127584341	C	A	29	10
127584549	T	G	27	15
127584572	C	T	29	20
127584576	G	A	29	20
127584614	C	T	30	13
127584631	G	A	29	15
127584687	G	C	31	30
127584688	C	A	31	30
127584914	G	A	31	16
127585027	C	A	29	12
127585053	C	T	24	6

Table S7: FANK1 promoter region variants that appeared "real" after visual inspection of .bam files in IGV and re-calling of variants (which were originally called with VarScan) with GATK. All variants are seen as heterozygotes, therefore allele counts equate to the number of individuals carrying the alternative variant.

Window (bp, GRCh38)	L	M_st	M_p	STB_p	Probable variant position	Variant (REF/ALT)	rsID	VUR AAF (n= 32)	ALSPAC AAF (n=1106)	Gene
124841200-124841319	1	1	1	6.52x10-4	124841267	C/A	rs533072490	0.04	0.0	3 kb upstream FAM175B
125655040-125655159	1	1	1	4.43x10-4	125655088	A/T	rs183471950	0.0468	0.0016	TEX36
125679640-125679759	1	1	1	3.83x10-4	125679648	G/A	rs541040960	0.0313	0.0	TEX36
127059640-127059759	1	1	1	1.25x10-4	127059696	A/AT	rs551248465	0.0468	5.7x10-4	DOCK1
128329480-128329599	1	1	1	5.58x10-4	128329528	A/G	rs146303503	0.0468	0.0012	10 kb downstream LINC01163
128770720-128770839	1	1	1	8.88x10-4	128770839	G/A	-	0.0313	0	400 kb downstream LINC01163
128929840-128929959	1	1	1	6.51x10-4	128929870	G/A	rs182100352	0.0313	0	500 kb upstream MGMT

Table S8: The 7 out of the top 10 TASER results that were considered reliable, with MAF threshold 0.05 where L = number of variants “seen” in a particular 120 bp window; M_st = number of variants passing screening and threshold procedures; M_p = probable “true” variants; New-STB_p = Burden statistic p value. VUR and ALSPAC MAFs calculated from read counts at the variant position as determined by inspection of the appropriate “read-count” input file.
